# Still not sterile: viability-based assessment of the skin microbiome following pre-surgical application of a broad-spectrum antiseptic reveals transient pathogen enrichment and long-term recovery

**DOI:** 10.1128/spectrum.02873-24

**Published:** 2025-04-10

**Authors:** Elizabeth C. Townsend, Kayla Xu, Karinda De La Cruz, Lynda Huang, Shelby Sandstrom, Delanie Arend, Owen Gromek, John Scarborough, Anna Huttenlocher, Angela L. F. Gibson, Lindsay R. Kalan

**Affiliations:** 1 Department of Medical Microbiology and Immunology, University of Wisconsin School of Medicine and Public Health 5232, Madison, Wisconsin, USA; 2 Microbiology Doctoral Training Program, University of Wisconsin-Madison 5228, Madison, Wisconsin, USA; 3 Medical Scientist Training Program, University of Wisconsin School of Medicine and Public Health 5232 , Madison, Wisconsin, USA; 4 Department of Bacteriology, University of Wisconsin-Madison 205263, Madison, Wisconsin, USA; 5 Department of Biochemistry and Biomedical Sciences, McMaster University 152940, Hamilton, Ontario, Canada; 6 M.G. DeGroote Institute for Infectious Disease Research, David Bradley Centre for Antibiotic Discovery, Department of Biochemistry and Biomedical Sciences, McMaster University 536887, Hamilton, Ontario, Canada; 7 Department of Surgery, University of Wisconsin School of Medicine and Public Health 167468, Madison, Wisconsin, USA; 8 Division of Allergy, Immunology and Rheumatology, Department of Pediatrics, University of Wisconsin School of Medicine and Public Health 200763, Madison, Wisconsin, USA; 9 Division of Infectious Disease, Department of Medicine, University of Wisconsin School of Medicine and Public Health 206023 , Madison, Wisconsin, USA; Duke University, Durham, North Carolina, USA

**Keywords:** skin microbiome, antiseptic, viability PCR

## Abstract

Broad-spectrum antiseptics such as chlorhexidine gluconate (CHG) have widespread use as pre-surgical tools to lower skin microbial burden and reduce the risk of surgical site infection. However, the short- and long-term effects of CHG on healthy skin microbial communities remain undefined due to the confounding effects of CHG binding with persistent bacterial DNA on the skin surface. Here, we aim to accurately characterize the immediate and long-term impact of pre-surgical preparation with CHG-based antiseptics on the human skin microbiome. Twenty-eight patients undergoing elective surgeries were enrolled. Swabs of the surgical site and a control site skin microbiome were collected at multiple time points before and up to 2 weeks after surgery. A propidium monoazide (PMAxx)-based viability assay was optimized to selectively evaluate DNA from live microbes in complex skin microbial communities with viability-qPCR and viable 16S ribosomal RNA gene profiling. Pre-operative CHG induces a measurable reduction in the viable microbial bioburden at the surgical site. On the day of surgery, surgical sites displayed a significant increase in the relative abundance of several SSI-associated bacterial genera including *Acinetobacter, Bacillus, Escherichia-Shigella,* and *Pseudomonas* compared to baseline. *Bacillus* species isolated from subjects at baseline also demonstrate resistance to CHG with minimum inhibitory concentrations exceeding 1,000 µg/mL. Although there are major skin microbiome shifts upon exposure to CHG, we also find that these shifts are largely transient. For the majority of individuals, skin microbial bioburden and community structure recover to near baseline by post-surgical follow-up.

**IMPORTANCE:**

Surgical site infections continue to occur despite widespread adoption of surgical antiseptics. Before surgery, patients often wash their whole body multiple times with chlorhexidine gluconate (CHG)-based antiseptic soap and have CHG applied to the surgical site in the operating room. However, the effects of CHG antiseptics on the healthy skin microbiome are undefined due to CHG persisting and binding DNA from dead cells on the skin. We optimized a viability assay to selectively target DNA from live microbes on the skin before and after exposure to CHG. Our findings demonstrate that pre-surgical application of CHG significantly reduces the bioburden on skin; however, potentially pathogenic bacteria remain. Post-surgery, the skin microbiome eventually recovers to resemble its pre-CHG exposed state. Collectively, these findings identify tangible avenues for improving antiseptic formulations and further support that the skin microbiome is viable, stable, and resilient to chemical perturbation.

## INTRODUCTION

Surgical site infections (SSI) pose a substantial burden to affected patients and the healthcare system. Despite widespread adoption of broad-spectrum antiseptics to reduce the skin microbial bioburden at the time of surgery, SSI still occur. Approximately 0.5% to 3% of all surgical patients will experience an infection at or adjacent to their surgical incision (1, 2). Collectively, these infections pose an additional $3.5–$10 billion in annual healthcare costs due to prolonged hospital stays, additional diagnostic tests, procedures and operations, and increased utilization of outpatient resources (3, 4). To prevent SSI, antiseptics intentionally reduce skin microbial bioburden at the time of surgery, but in the process disrupt the skin’s naturally occurring microbial communities. Defining the impact of these antiseptics on the skin microbiome across various surgeries and surgical sites is needed.

The skin microbiome comprises complex microecosystems of bacteria, fungi, and viruses (5–7). Under normal health conditions, commensal skin microbes promote skin health by enhancing barrier function and preventing pathogen overgrowth through competitive interactions and niche exclusion (5, 7, 8). There is currently a major gap in our understanding of the differential impacts of antiseptics on skin microbial communities, including potential selective pressures promoting antiseptic resistance. How and the degree to which these complex skin microbial communities recover following antiseptic application is also yet to be characterized.

Standard pre-surgical preparations often involve bathing with 4% chlorhexidine gluconate (CHG) soap the night before and morning of surgery, followed by local application of CHG (e.g., ChloraPrep) to the surgical site (9–12). CHG and other topical antiseptics are validated for clinical use through demonstrating a significant reduction in the culturable microbial bioburden for up to 48 hours post-application (13–20). However, the impact of broad-spectrum antiseptics like CHG on healthy skin microbial communities remains unknown since previous sequencing-based efforts to quantify and characterize their impact on skin microbial communities have yielded mixed results. While some demonstrate a reduction in the microbial diversity following CHG exposure (21), most studies conclude that no significant change in skin microbial community structure or bioburden occurs (22–25). This discrepancy between culture-dependent and -independent studies is likely due to CHG’s ability to bind persistent bacterial DNA, particularly DNA from recently lysed cells, to the surface of the skin (22, 26). This subsequently confounds sequencing-based analyses that do not account for the viability of sampled microbes. Further, this underscores the pressing need for establishing a method capable of accurately evaluating live microorganisms on the skin, particularly after a toxic (e.g., antiseptic) exposure, via DNA sequencing-based technologies.

With this work, we aim to characterize the immediate and long-term impacts of CHG antiseptic on viable skin microbial bioburden and community composition in patients undergoing elective surgery. To selectively evaluate DNA from live microorganisms within the skin microbiome pre- and post-antiseptic exposure, we optimized a propidium monoazide (PMAxx)-based viability assay. Contrary to a prior work concluding that there are few viable bacteria on the skin (27), we show that most bacterial cells collected from skin microbiome samples are viable. Overall, we confirm pre-operative preparation with CHG effectively reduces viable microbial bioburden at the surgical site. Concurrently, we see enrichment of potentially pathogenic taxa on the day of surgery. We further demonstrate that these shifts are transient, with the majority of individuals’ skin microbiomes recovering, both in absolute abundance and microbial community structure, by their post-surgical clinic follow-up. *Bacillus* and *Enterococcus* species isolated from the skin of these surgical patients also display increased resistance to CHG. Collectively, these findings identify avenues to spur the development of improved pre-surgical protocols and broaden our understanding of skin microbiome dynamics.

## MATERIALS AND METHODS

### Subject identification and enrollment

Adults 18 years or older undergoing elective surgery were enrolled from the UW-Health General Surgery Clinic. Inclusion criteria for the study were as follows: i) person 18 years or older, ii) undergoing elective surgery requiring preoperative CHG, iii) surgical wounds anticipated to be classified as clean, clean-contaminated, or contaminated, and iv) able to provide written and verbal consent. Exclusion criteria were as follows: i) emergent surgery or surgery due to trauma, ii) recent burn encompassing more than 30% of the total body surface area, iii) surgical wounds anticipated to be classified as dirty-infected, iv) known allergy to chlorhexidine, or v) administered antibiotics (not including those for surgical prophylaxis) within 2 weeks of enrollment or the surgery. Information related to pre-surgical assessments, surgery, post-surgical care, following post-operative outpatient evaluations, and patient co-morbidities was extracted from the medical records. If a subject developed a surgical site infection or other surgical site occurrences developed, information related to its assessment and care was also recorded.

### Survey of subject home and work environment

To gauge environmental factors that may influence subject skin microbial communities, subjects completed a survey to collect information on their skin health, hygiene habits, and home and work environments. This survey was administered by study staff at the initial clinic visit following enrollment. Questions pertained to the following: i) select skin and medical history that may not be apparent in their medical chart (e.g., do they consider their skin to generally be oily, dry, or a combination), ii) personal hygiene habits (e.g., number of showers per week), iii) home environment (e.g., do they live in an urban, suburban, or rural setting), iv) work environment (e.g., do they work in a health care setting, and v) exposure to animals (e.g., do they live with cats, dogs, or other animals).

### Pre-surgical preparation

Subjects were provided with a 4% CHG soap (Hibiclens; Mölnlycke Health Care, Norcross, Georgia) to take home and shower with the night prior and morning of their surgery following the instructions outlined in the UW-Health pre-surgical guidelines (9). When patients arrived in the pre-operative waiting area, patients self-reported the number of showers/baths completed with CHG prior to their surgery and whether they used other hygiene products (e.g., shampoos and body wash) for these showers. In the operating room, a CHG-based antiseptic was applied according to surgical guidelines. In general, a 26 mL applicator of 2% CHG with 70% isopropanol (ChloraPrep; Becton, Dickinson and Company (BD), Franklin Lakes, NJ) was used following manufacturers’ instructions. The CHG solution was left to air-dry for at least 3 minutes prior to the sample collection and subsequent incision.

### Sample collection

**Fig 1:**
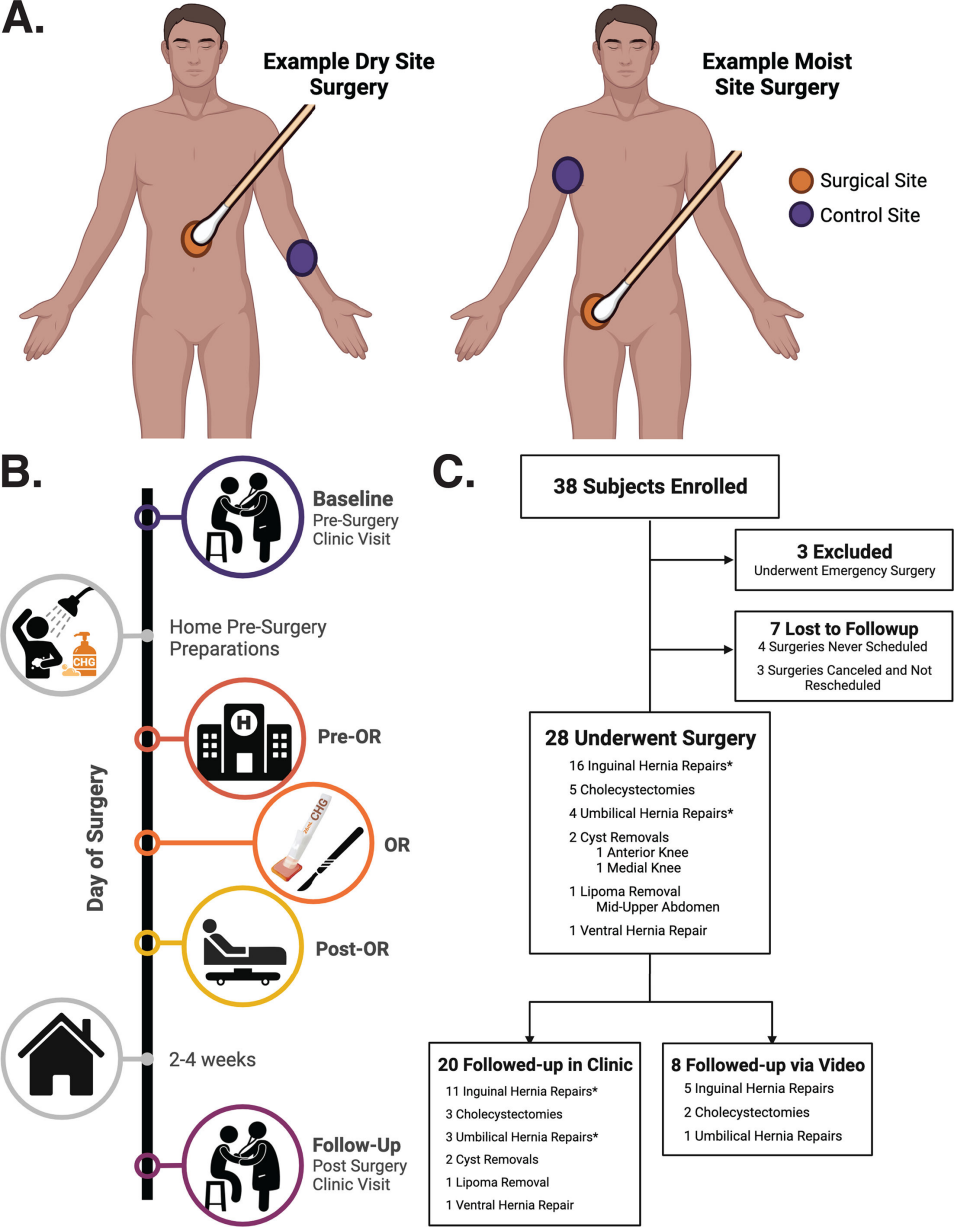
Study design. (**A**)Skin microbiome sampling locations for dry skin site surgeries (e.g., central or upper abdomen and knee; left image) and moist skin site surgeries (e.g., lower abdomen to inguinal crease or the umbilicus; right image). (**B**)Sample collection timeline. Collections include the following: i) at the pre-surgery evaluation clinic visit; ii) on the day of surgery in the pre-operative care unit, in the operating room after CHG was applied and allowed to dry for 3 minutes yet prior to surgical incision, and in the post-anesthesia care unit before discharge; and iii) at the post-surgery clinic visit. (**C**)Flow chart illustrating the number of enrolled subjects who were excluded or underwent elective surgery and had their post-surgery clinic visit in person or over video. * Indicates one subject who underwent simultaneous inguinal and umbilical hernia repair. For this subject, both surgery sites were sampled at each time point.

Swabs of the skin microbiome were collected from the intended surgical site (pre-surgery)/around the incision (after surgery) and a similar control site that was not anticipated to receive CHG application in the operating room. The armpit served as the control site for surgeries at moist body sites (e.g., inguinal or umbilical hernia repairs), and the volar forearm served as the control site for surgeries at dry body sites (e.g., cholecystectomies or ventral hernia repairs). If CHG or another antiseptic had been applied to the volar forearm for IV insertion, the contralateral forearm was used. Fig. 1A illustrates example sampling locations. Swabs were collected from both the surgical site and a control site at multiple time points before and after surgery (Fig. 1B).

Swabs designated for DNA extraction were placed in 155 µL of 1% bovine serum albumin (BSA) in 1 x phosphate-buffered saline (PBS) and were stored at 4°C for less than 2 hours before processing for selective detection of DNA from viable organisms (per below) and eventual DNA extraction. Swabs designated for microbial culture were placed in 1 mL of liquid Amies (Copan Diagnostics Inc., Murrieta, CA). Culture swabs were stored at 4°C for less than 2 hours before being processed for microbial culture.

### Microbial culture and bacterial isolate identification

Swabs designated for microbial culture were spun-down using DNA IQ Spin Baskets (Promega, Madison, WI). A portion of each sample was serially diluted with 1X PBS and plated onto tryptic soy agar (TSA) with 5% sheep blood (BBL, Sparks, MD), brain–heart infusion (BHI) agar, and BHI agar with 1% Tween 80 and 50 mg/L mupirocin for quantitative bacterial culture. Plates were incubated at 35°C overnight. To isolate culturable bacteria, colonies with a distinct morphology were isolated and incubated at 35°C overnight on the same media as the original culture or BHI with Tween without mupirocin, and then single colonies were inoculated into liquid TSB, BHI broth, or BHI broth with 1% Tween for overnight incubation. To identify each bacterial isolate, a portion of the overnight culture was subjected to DNA extraction, and Sanger sequencing (Functional Biosciences, Madison, WI) of the bacterial 16S ribosomal RNA gene was performed. The remaining portion of the isolate culture was stored in glycerol at −80°C.

### Measuring CHG resistance in bacterial isolates

Select bacterial isolates collected from surgical subjects during their baseline and follow-up clinic visits were evaluated for resistance to CHG via a disk diffusion minimum inhibitory concentration (MIC) assay. Isolates were selected for MIC testing with the intent to evaluate a representative range of bacterial species and, when possible, to have multiple isolates of the same species from different subjects. Isolates were grown overnight in BHI with 1% Tween 80 liquid media at 37°C. Overnight cultures were diluted with 1X PBS using optical density readings to achieve 1 × 10^8^ bacterial colony-forming units/mL. Diluted cultures were plated to create a bacterial lawn on BHI agar with 1% Tween 80, and plates were dried for 5–10 minutes prior to the placement of 6 mm Whatman Antibiotic Assay Disc (Cytiva, Buckinghamshire, United Kingdom). Then, 2% CHG in 70% isopropanol (ChloraPrep; BD) was serially diluted with 1X PBS, and then 15 µL of each dilution was added to a respective disk to achieve a concentration range of 1–2,000 µg/mL CHG. A disk with 1X PBS served as the negative control. Plates were then incubated for 18 hours overnight at 37°C. For each isolate, the disk with the smallest zone of inhibition, either just under the disk or extending no more than 1–3 millimeters beyond the disk, was considered the MIC of CHG. All isolates were tested in duplicate, and the average of the replicate MICs was calculated.

### Selective detection of DNA from viable microorganisms

**Fig 2:**
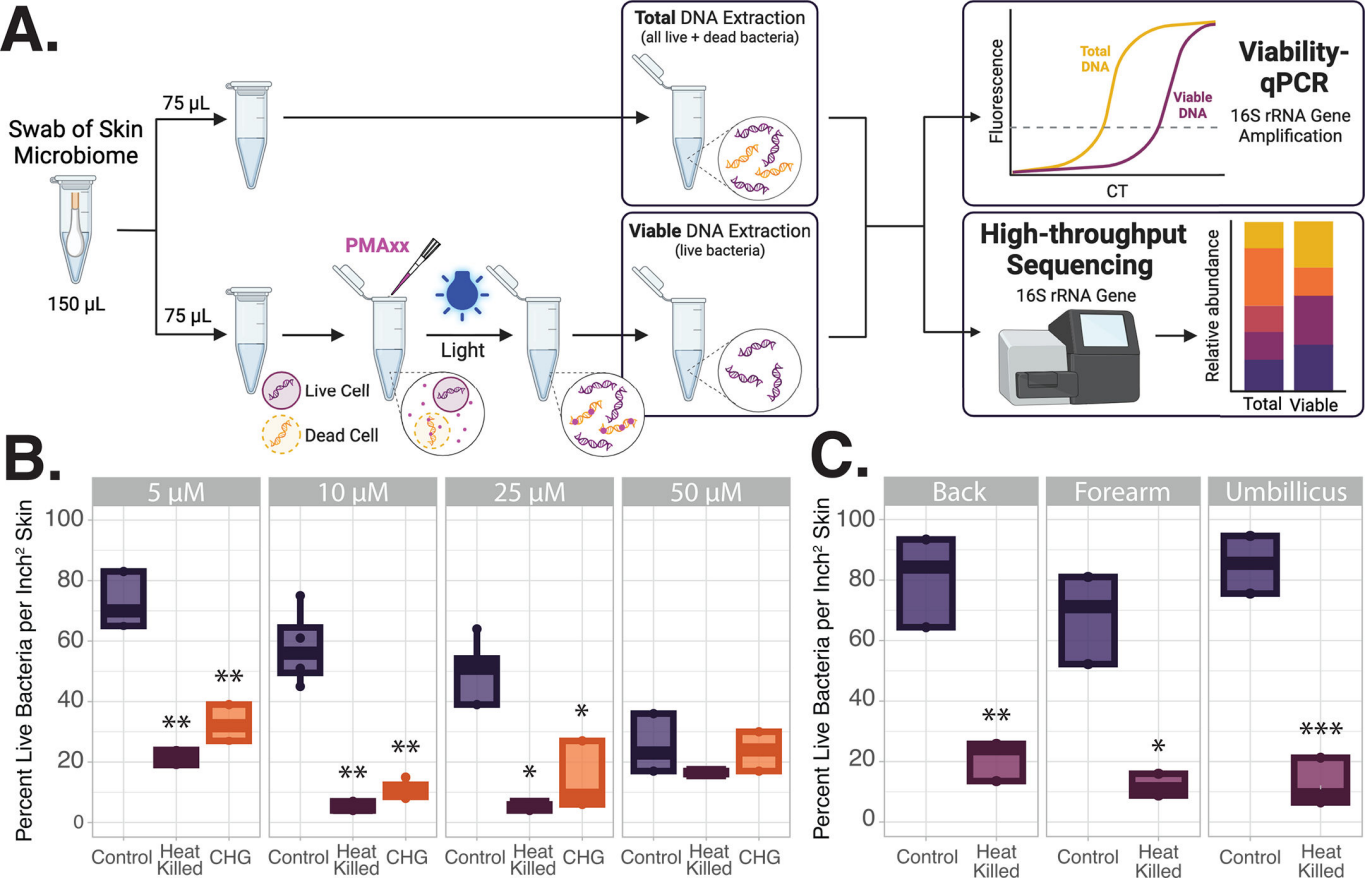
The optimal PMAxx concentration of 0 µM is used for selective evaluation of live bacteria within skin microbial communities. (**A**) Diagram of sample processing. Skin microbiome samples were split. Half of each sample was treated with PMAxx, which irreversibly binds to free DNA or DNA within compromised cells and prevents it from being separated for PCR amplification or sequencing. This allows for selective amplification of only DNA from viable bacteria. The remaining half of the sample remained untreated to evaluate the DNA from both live and dead bacteria (total DNA). Total and viable bioburden was evaluated via quantitative PCR of the bacterial 16S ribosomal RNA gene (viability–qPCR). Viable and total microbial communities were further evaluated via high-throughput sequencing. (**B**) Four different concentrations of PMAxx were evaluated for accurate quantification of viable and total bacterial bioburden on the volar forearm. Swabs of the forearm microbiome were collected from volunteers; one sample was heat-killed at 95°C for 10 minutes, and one sample was collected following application of CHG (*n* = 4 for 10 µM, *n* = 3 for remaining concentrations). Bioburden was quantified via viability–qPCR. The percentage of live bacteria was calculated by dividing the number of viable bacteria in the sample by the total number of bacteria. The percent live bacteria in heat-killed samples and samples from CHG-treated skin were compared to the control group by *t*-test with Welch’s correction. (**C**) Ten micromoles of PMAxx accurately distinguishes control and heat-killed microbial communities from multiple skin sites. Swabs from the back (sebaceous site), volar forearm (dry), or umbilicus (moist) were collected from volunteers. The plot displays the percent live bacteria in control and heat-killed microbiome samples from the back, forearm, and umbilicus (*n* = 3). Groups compared with via *t*-test with Welch's correction. * *P*-value < 0.05; ** *P*-value < 0.01; *** *P*-value < 0.001; **** *P*-value < 0.0001.

Swabs collected in 1% BSA in PBS were spun-down using DNA IQ Spin Baskets (Promega, Madison, WI), and each sample was split into two equal 75 µL portions. To quantify and sequence the DNA from all (live and dead) members of the skin microbial communities, one portion of each sample was placed directly in −20°C storage. To selectively quantify and sequence DNA from only live microbes within skin microbial communities, the other portion of each sample was processed with a modified propidium monoazide (PMAxx, Biotium, Fremont, CA)-based viability assay (Fig. 2A). PMAxx is a photoreactive molecule that when light-activated covalently binds to DNA (28). PMAxx is also unable to pass through intact cell membranes. Thus, when activated, PMAxx will irreversibly bind to only free DNA and DNA within compromised (dead) cells. PMAxx-modified DNA is unable to be separated for PCR amplification or sequencing, allowing for selective amplification and sequencing of only the unmodified DNA from intact (live) cells. All steps involving PMAxx were performed in a dark room. PMAxx was added to achieve a final concentration of 10 µM in each sample. Samples were shaken at room temperature for 10 minutes, exposed to blue light for 15 minutes via the PMA-Lite LED Photolysis Device (Biotium, Fremont, CA), and then spun at 5,000 *g* for 10 minutes. Both the PMAxx-treated portion and untreated portion of each sample were stored at −20°C before DNA extraction.

### DNA extraction, library construction, and sequencing

DNA extraction was performed as previously described with minor modifications (29). Briefly, 300 µL of the yeast cell lysis solution (from Epicentre MasterPure Yeast DNA Purification kit), 0.3 µL of 31,500 U/µL ReadyLyse Lysozyme solution (Epicentre, Lucigen, Middleton, WI), 5 µL of 1 mg/mL mutanolysin (M9901, Sigma-Aldrich, St. Louis, MO), and 1.5 µL of 5 mg/mL lysostaphin (L7386, Sigma-Aldrich, St. Louis, MO) were added to 150 µL of the swab liquid before incubation for 1 hour at 37°C with shaking. Samples were transferred to a 2 mL tube with 0.5 mm glass beads (Qiagen, Germantown, Maryland) and bead-beat for 10 minutes at maximum speed on a Vortex-Genie 2 (Scientific Industries, Bohemia, NY), followed by a 30 minute incubation at 65°C with shaking and 5 minute incubation on ice. The sample was spun-down at 10,000 rcf for 1 minute, and the supernatant was added to 150 µL of the protein precipitation reagent (Epicentre, Lucigen, Middleton, WI) and vortexed for 10 seconds. Samples were spun-down at maximum speed (~21,000 rcf) and allowed to incubate at RT for 5 minutes. The resulting supernatant was mixed with 500 µL isopropanol and applied to a column from the PureLink Genomic DNA Mini Kit (Invitrogen, Waltham, MA) for DNA purification using the recommended protocol.

The viability quantitative polymerase chain reaction (viability-qPCR) was performed to determine the amount of DNA from viable bacteria (treated with PMAxx) and total DNA from both live and dead bacteria (non-PMAxx treated portion) of each sample. In short, 1 µL of the extracted DNA was added to a reaction mix containing 5 µL TaqMan Fast Advanced 2X Master Mix (Applied Biosystems, Waltham, MA), 0.5 µL TaqMan 16S 20X Gene Expression Assay with FAM (Applied Biosystems), and 3.5 µL PCR pure water. Samples were run for 40 thermocycles on the QuantStudio 7 Flex Real-Time PCR System (Applied Biosystems). Sample DNA concentrations were determined based on a standard curve of 0.015 to 15,000 pg/µL DNA extracted from *Escherichia coli* (ATCC 1496).

16S rRNA gene V3–V4 region amplicon libraries were constructed using a dual-indexing method at the University of Wisconsin Biotechnology Center and sequenced on a MiSeq with a 2 × 300 bp run format (Illumina, San Diego, CA). Reagent-only negative controls were carried out through the DNA extraction and sequencing process. A 20-Strain Staggered Mix Genomic Material (ATCC, Manassas, VA) served as a positive sequencing control.

### Sequence analysis

The QIIME2 (30) environment was used to process DNA-based 16S rRNA gene amplicon data. Paired-end reads were trimmed, quality-filtered, and merged into amplicon sequence variants (ASVs) using DADA2. Taxonomy was assigned to ASVs using a naive Bayes classifier pre-trained on full-length 16S rRNA gene 99% OTU reference sequences from the SILVA SSU database (release 138). Using the qiime2R package, data were imported into RStudio (version 1.4.1106) running R (version 4.2.1) for further analysis using the phyloseq package (31). Negative DNA extraction and sequencing controls were evaluated and removed from all samples based on absolute read count and ASV distribution in true patient samples. Abundances were normalized proportionally to total reads per sample. Plots were produced using the ggplot2 package. Taxa below 0.5% relative abundance were pooled into an “Other” category. Univariate and/or multivariate permutational multivariate analysis of variance (PERMANOVA) were used to evaluate associations between microbial community compositions and subject features. Each PERMANOVA was run considering the marginal effects of terms with 9,999 permutations using Adonis2 in the vegan r package (32). ASVs from the *Staphylococcus* genus were aligned to the BLAST nucleotide database (2.14.1+) to assign probable species. MAASLIN2 (33) was utilized to identify significant differences in taxa abundance between various groups.

### Statistical analyses

Most statistical analyses were conducted in R Studio running R (version 4.2.1). Comparisons of subject demographics and comorbidities between patient groups were analyzed via Prism (version 9.2.0).

## RESULTS

### Subjects

Thirty-eight subjects undergoing elective surgeries were enrolled (Fig. 1C, Table 1). Ten individuals were excluded because the surgery was not performed as planned. The remaining individuals ranged in age from 30 to 81 years (mean 58 ± 15 years). The proportion of subjects with comorbidities and self-reported home and work environmental conditions were not significantly different between the two surgical sub-groups (Table S1). Twenty-four of the 28 subjects (85.7%) received antibiotic prophylaxis with a single dose of intravenous cefazolin the day of surgery. Twenty of the subjects came to the clinic for their follow-up 2–4 weeks post-surgery, while eight conducted their follow-up over video, and the final “Follow-up” skin microbiome samples were subsequently unable to be collected (Fig. 1C).

**TABLE 1:** Subject demographics, including average age, gender, self-reported race, and ethnicity for the 38 enrolled subjects^*a*^

	All subjects	Excluded or lost to follow-up	All subjects who underwent surgery	Dry site surgeries	Moist site surgeries
Basic Demographics					
Number of subjects	38	10	28	9	19
Age ±SD	57.3 ± 15.7	54.4 ± 18.26	58.4 ± 14.9	58.7 ± 15.8	58.2 ± 14.9
Male	24 (63.1%)	3 (30%)^*b*^	21 (75%)^*b*^	4 (44.4%)^*c*^	17 (89.5%)^*c*^
Race %					
White	37 (97.3%)	10 (100%)	27 (96.4%)	9 (100%)	18 (94.7%)
Mixed race: American Indian or Alaskan Native and White	1 (2.6 %)	0	1 (3.6%)	0	1 (5.3%)
Ethnicity					
Not Hispanic or Latino	34 (89.4%)	9 (90%)	26 (89.3%)	7 (77.8%)	18 (94.7%)
Hispanic or Latino	3 (7.9%)	1 (10%)	2 (7.1%)	1 (11.1%)	1 (5.3%)
Middle Eastern	1 (2.6%)	0	1 (3.6 %)	1 (11.1%)	0

^*a*^ Demographics are also broken down for the 10 subjects who were excluded or lost to follow-up, the 28 subjects who ultimately underwent surgery collectively, as well as the 9 and 19 subjects, respectively, who underwent surgeries at dry or moist body sites.

^*b*^ *P*-value < 0.05 for subjects who underwent surgery vs excluded, Fishers exact t-test.

^*c*^ *P*-value < 0.05 for subjects who underwent moist skin site surgeries vs dry site surgeries, Fishers exact t-test.

### Selectively evaluating viable skin microorganisms

To selectively evaluate DNA from live bacteria within skin microbial communities, prior to DNA extraction, samples were split, with half being treated with propidium monoazide (PMAxx; Fig. 2A). PMAxx is unable to pass through intact (live) cells, and thus when light activated, it will irreversibly bind to only free DNA and DNA within compromised (dead) cells (28). This PMAxx-modified DNA is unable to be separated, allowing for selective amplification and sequencing of only the unmodified DNA from live cells. To evaluate the total DNA from both live and dead bacteria, the remaining sample half remained untreated. To determine the optimal PMAxx concentration for complex but low biomass skin samples, swabs of the skin microbiome were first collected from the volar forearm of healthy volunteers (Fig. 2B). Overall, 10 µM PMAxx performed well at accurately stratifying the higher percentage of DNA from live bacteria in the control sample (58±13%) compared to the heat-killed control (5±1%) or from skin treated with CHG antiseptic (11±3%; both *P*-values < 0.01 both vs control, *t*-tests with Welch’s correction). The comparatively low percentage of live bacteria in the control sample treated with 50 µM PMAxx (25%) suggests that excessive PMAxx in the solution is cytotoxic.

Microbial bioburden and community structure vary across skin sites; 10 µM PMAxx was also able to accurately distinguish control and heat-killed microbial communities from multiple skin sites (all *P*-values < 0.05, *t*-test with Welch's correction) Fig. 2C. Together, Fig. 2B and C highlight the importance of identifying the appropriate PMAxx concentration for the intended study community type (27). These findings also contradict the recent claim that under normal circumstances, the majority of bacterial DNA on the skin is from deceased microbes. Across all experiments and sampled body sites, the percentage of live bacteria on unperturbed skin ranges from 43% to 86% for dry body sites (95% CI of 58%–65%) and from 43% to 99% for moist sites (95% CI of 70% to 76%; Table S2; Fig. 2B and C; Fig. S1A).

### Pre-Surgical preparation with CHG reduces both total and viable microbial bioburden the day of surgery

To determine the impact of pre-surgical preparation with CHG on skin microbial bioburden, swabs of the skin were collected from each subject’s intended surgical site and a control site during their initial clinical visit, multiple times on the day of surgery, and on their follow-up clinic visit (Fig. 1B). Swabs were collected from the surgical site during recovery in the post-operation care unit for quantitative bacterial culture. On the day of surgery, 5/28 subjects had culturable bacteria (Fig. S1B).

**Fig 3:**
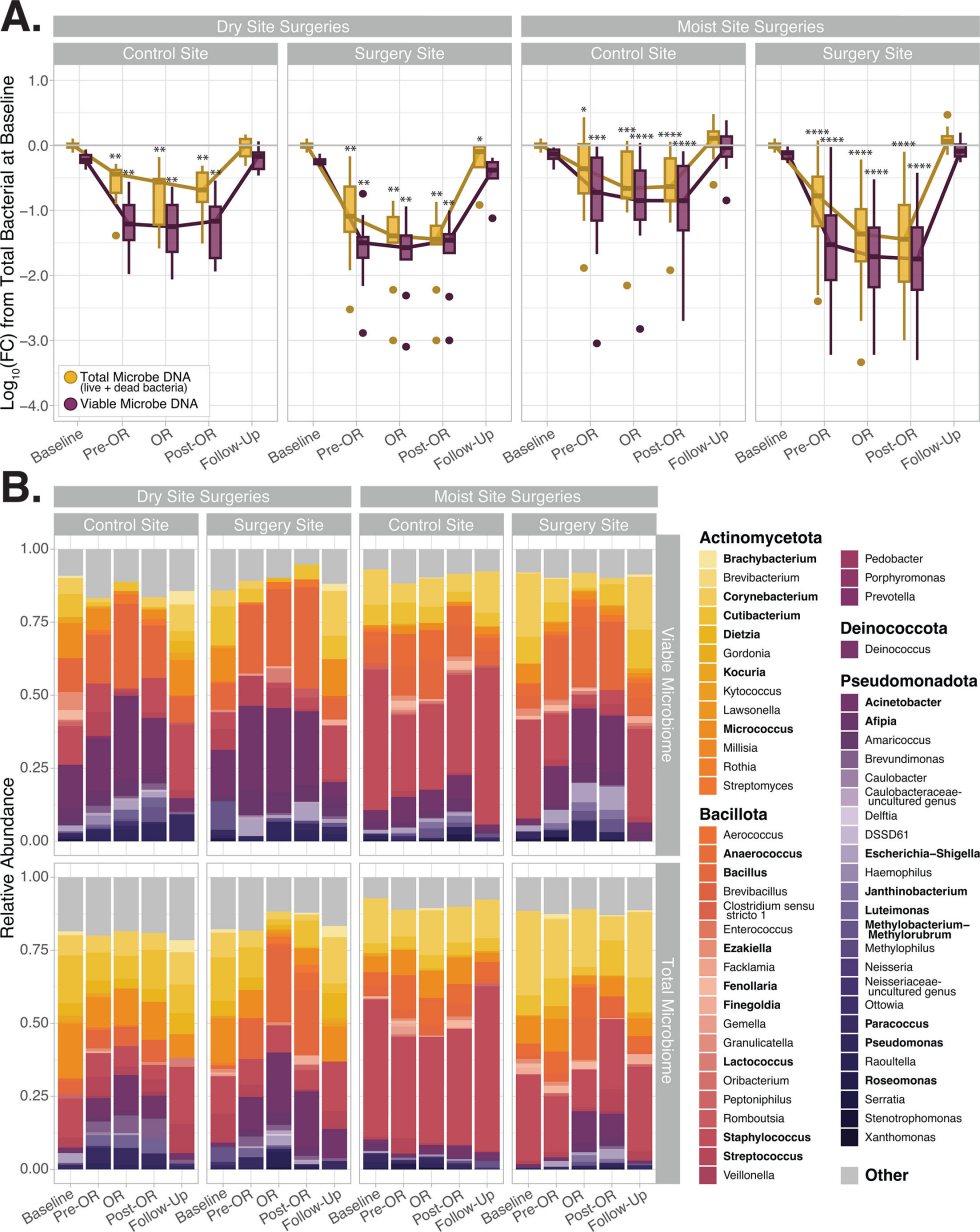
Viable and total subject skin microbial communities over time. (**A**) Exposure to CHG induces a significant reduction in both total (yellow) and viable (purple) microbial communities the day of surgery (Pre-OR, OR, and Post-OR timepoints). Data displayed as the log_10_(fold change) from each subject’s total bacterial bioburden at baseline. *P*-values represent Wilcoxon matched pairs ranked tests between the fold change in total or viable bioburden at a later time point vs the respective total or viable microbial bioburden at baseline. More details on the viable and total bioburden along with additional statistical comparisons are provided in Table S3. Details on the percentage of live bacteria in these samples are provided in Fig. S1. (**B**) Plots display the average relative abundance of each genus at the surgical or control site at each time point for all the subjects who underwent moist or dry surgeries. Average genera relative abundance within the viable and total (live +dead) microbial communities are shown in the top and bottom rows, respectively. Bolded genera are those comprising at least 30% of the microbial community in at least one sample. Other represents taxa <0.5% in proportion.

Viability-qPCR of the bacterial 16S rRNA gene was used to quantify total and viable bacterial bioburden across all time points (Fig. 3A). Due to high interindividual variations in baseline microbial bioburden, the log_10_(fold change) from each subject’s baseline surgical site or control site bioburden was calculated (Fig. 3A; Table S3). Showering with a CHG soap induced a roughly 1.25 log_10_-fold reduction in the viable microbiome at both moist and dry surgical sites (*P*-values < 0.01 pre-OR vs baseline). This corresponded with a 0.84 log_10_-fold reduction in total microbial DNA (*P*-value < 0.01 pre-OR vs baseline). Application of CHG to the surgical site just before incision further reduced the viable microbial bioburden by an additional 0.19 log_10_ and 0.04 log at moist and dry surgical sites, respectively (all *P*-values < 0.01 OR vs baseline). In general, moist surgical sites displayed greater reduction in microbial communities at the OR and post-OR timepoints than dry surgical sites, likely due to greater baseline microbial bioburden. Control sites exhibited roughly 0.75 and 0.43 log_10_-fold reduction in their viable and total microbial bioburden, respectively, at the pre-OR sample collection (all *P*-values < 0.01). While showering alone can reduce the microbial bioburden, the decline observed here is likely in large part due to exposure of control skin sites to CHG soap during the pre-operative showers. Further details on the percentage of DNA from live bacteria at each time point are illustrated in Fig. S1A.

### Skin microbial communities before, during, and after the day of surgery

To characterize the impact of CHG antiseptic on skin microbial community structure, both viable and total bacterial DNA sample portions were subjected to 16S rRNA profiling. *Staphylococcus, Corynebacterium, Cutibacterium, Acinetobacter,* and *Bacillus* genera comprised the main taxa in viable and total samples across all sites (Fig. 3B). No significant differences were observed in viable or total microbiome structure in samples collected at baseline or at the post-surgical clinic follow-up (*P*-values > 0.05, PERMANOVA, Fig. S2A and E; Table S4). This similarity of patients’ viable and total microbiomes at baseline and their clinical follow-up confirms that our viability protocol works well in normal, baseline conditions selectively evaluating DNA from live microbes without overrepresenting groups of taxa from skin microbial communities. Viable microbial communities were significantly different than the total microbiome on the day of surgery for samples collected at the Pre-OR, OR, and Post-OR timepoints (all *P*-values < 0.001; Fig. S2B through D; Table S4). These differences are driven by an overrepresentation of residual DNA from *Corynebacterium*, *Micrococcus,* and *Staphylococcus* species on the day of surgery (Fig. S2F through H).

### Clinical features influencing the skin microbiome at baseline and on the day of surgery

Local skin physiology and the environment can influence the skin microbiome (7). To accurately address the primary aim of characterizing the effects of chlorhexidine antiseptic on viable skin microbial communities, we first identified the potential confounding effects of the subject and environmental factors on community composition. The body site of sample collection showed the largest differences in the microbiome composition, followed by sex of the participant (Fig. S3 and S4; Table S5). Antibiotic prophylaxis with cefazolin, even after accounting for sex and body site as confounding variables, was strongly associated with skin microbiome composition (*P*-value < 0.001 multivariate PERMANOVA). Patients who received antibiotic prophylaxis displayed a significant decrease in *Bacillus* and *Streptococcus* species at the surgical sites and a corresponding increase in *Pseudomonas* and *Afipia* (*P*-values < 0.05, FDR corrected q-values = 0.06, 0.13, 0.19, and 0.19 respectively, Fig. S4A through D; Table S6).

### Pre-surgical preparation with CHG promotes temporary shifts in the skin microbiome

**Fig 4:**
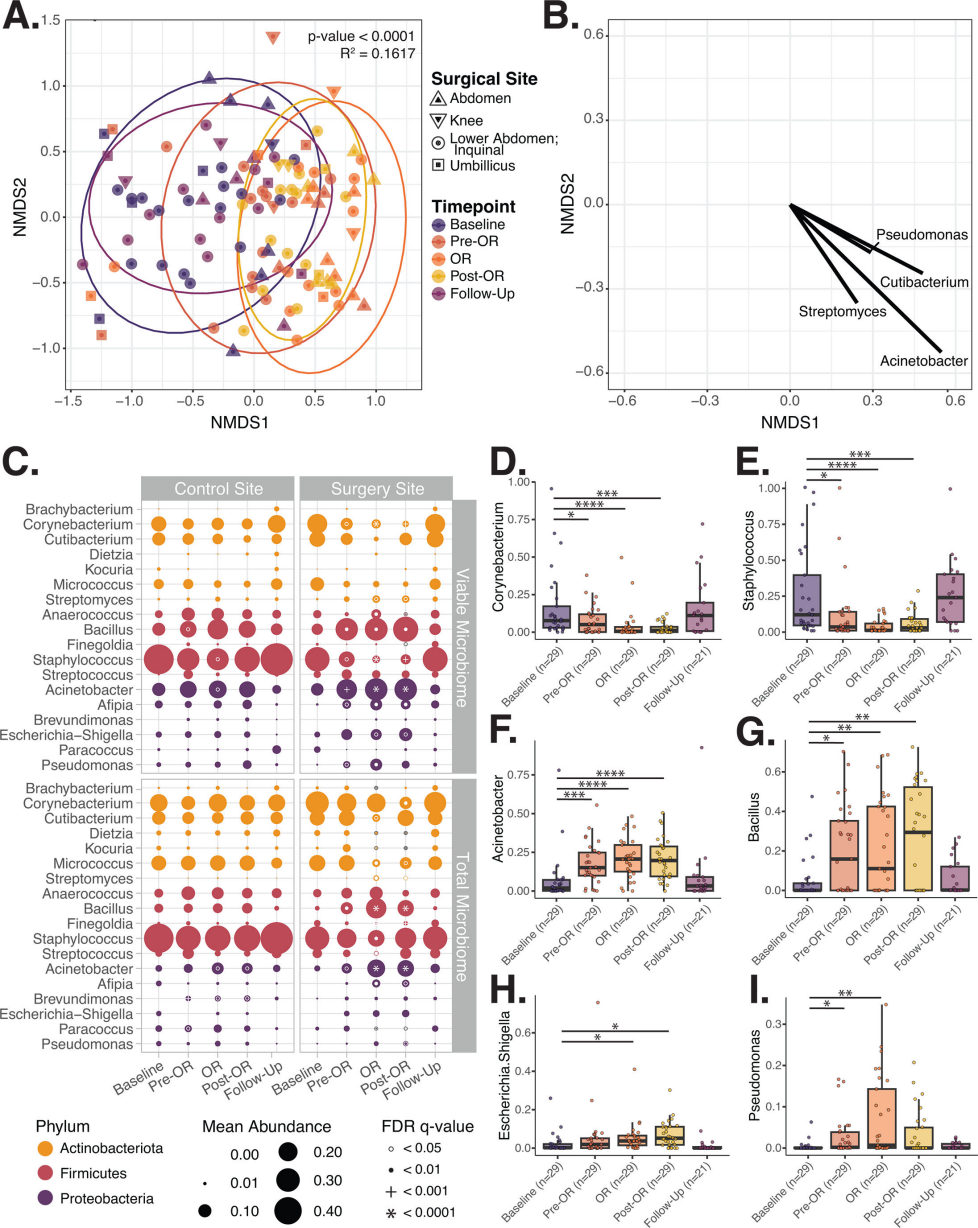
Pre-surgical application of chlorhexidine gluconate (CHG) antiseptic is associated with changes in the viable microbial community composition, particularly at the surgical site, on the day of surgery. (**A**) Bray–Curtis beta-diversity non-metric multi-dimensional scaling (NMDS) ordination displaying the significant shift in viable microbial community composition at subject surgery sites over time. Difference in the microbial community composition at each time point assessed via PERMANOVA with 9,999 permutations. (**B**) Vector plot displaying the taxa whose relative abundance significantly correlates with a point's position on the NMDS plot. Only taxa with a Spearman correlation *P*-value < 0.05 are shown. The longer the vector, the larger the Spearman rho. (**C**) Plot displaying the change in the average relative abundance of key taxa in the viable and total microbiome at control and surgical sites over time. Average taxa abundance across samples is indicated by the size of the point. Differential relative abundance of taxa at later time points compared to baseline was evaluated via MAASLIN2 accounting for the individual subject, subject gender, body site of sample collection, and use of pre-surgical antibiotic prophylaxis as random effects. White or gray circles, solid dots, plus sign, and asterisks indicate the degree of significance. (**D–I**) Relative abundance plots illustrating significant changes in key taxa within viable microbial communities at both moist and dry surgical sites over time. Points indicate the relative abundance of that taxa within a specific subject’s sample. Note; one subject underwent simultaneous umbilical and inguinal hernia repair. Both sites were sampled at all time points. Thus, the *n* = 29 at the baseline through Post-OR timepoints and *n* = 21 at follow-up, which is one more than the total number of subjects who underwent surgery and came for in-person follow-up, *n* = 28 and =20, respectively.

The longitudinal study design allowed us to track how the microbiome recovers following CHG application. After accounting for body site, gender, and antibiotic prophylaxis, the viable skin microbial community compositions at the surgical site on the day of surgery were significantly different than the baseline or follow-up clinical visit (*P*-value < 0.0001, multivariate PERMANOVA; Table S7). With univariate assessment, the time point of sample collection had the strongest association with viable community structure at the surgical site (*P*-value < 0.0001, univariate PERMANOVA; Fig. 4A; Table S7), followed by the body site of sample collection (*P*-value < 0.001; Fig. S5A).

On the day of surgery, surgical sites were enriched in the proportion of several bacterial genera associated with SSI, including *Acinetobacter, Bacillus, Escherichia-Shigella,* and *Pseudomonas*, compared to baseline communities (all FDR q-values <0.05, Pre-OR, OR or Post-OR vs Baseline, Fig. 4B through I; Fig. S5B through F; Table S8). These changes were accompanied by a corresponding reduction in commensal taxa relative abundance, particularly *Corynebacterium, Micrococcus,* and *Staphylococcus* (all FDR q-values <0.05, Pre-OR, OR or Post-OR vs Baseline). These trends were consistent across dry and moist body sites (Fig. S5 and S6; Table S8). Since *S. aureus* and *S. epidermidis* are notable SSI-associated pathogens, *Staphylococcus* ASVs were assigned probable species-level designation. This revealed that the reduction of *Staphylococcus* species on the day of surgery corresponded to reduced coagulase-negative staphylococci, particularly *S. epidermidis* (Fig. S5G). Increased *Acinetobacter* and *Bacillus* taxa on the day of surgery were primarily due to greater *A. lwoffii* and *B. subtilis,* respectively (Fig. S5H).

Both viable and total surgical site microbial communities from the baseline and follow-up clinic visits did not differ (*P*-value > 0.05, univariate PERMANOVA, Table S7). Collectively, the findings displayed in Fig. 4 and Fig. S6 demonstrate that pre-surgical preparation with CHG promotes significant shifts in microbial community composition at the surgical site. Both control and surgical site communities returned to near baseline levels after the patient returns home (*P*-value > 0.05, multivariate PERMANOVA, baseline vs follow-up; Fig. 4A; Table S7; Fig. S7A). However, at follow-up, subjects’ skin microbial communities were more similar to other individual baseline microbiomes than their own (*P*-values < 0.01, Wilcoxon paired signed rank test, Fig. S7B).

### Viability assay is needed to accurately detect skin microbial community changes following pre-surgical CHG

Compared to the viable microbiome, the shifts in total (DNA from both live and dead bacteria) microbiome community structure and taxa relative abundance were less pronounced and/or delayed and not noted until the OR or Post-OR sample collection (Fig. 4C; Fig. S6; Tables S7 and S8). Changes in the relative abundance of taxa noted in the viable community, including increases in *Escherichia* and *Pseudomonas*, were not detected when evaluating the total microbiome (Fig. 4C). These findings suggest if viability had not been assessed, overrepresentation of free DNA from abundant, lysed skin commensal taxa can mask true community shifts occurring.

### Pre-surgical preparation with CHG promotes smaller shifts in control site skin microbial communities

At control sites, both viable and total skin microbial communities were more similar over time (both *P*-values > 0.05, univariate PERMANOVAs; Fig. S6, S8 and S9; Table S7). On the day of surgery, control sites had subtle changes in taxonomic composition, including a minor rise in *Acinetobacter* and *Bacillus* relative abundance and minor decline in *Staphylococcus* (all FDR q-values <0.05, OR, Pre-OR, and OR vs Baseline; Fig. S8C through E). Additionally, there were no significant changes in the Shannon alpha diversity of total or viable bacteria within control or surgical site samples over the time course of sample collection (Fig. S9).

### *Bacillus* and *Enterococcus* spp. isolated from surgical patients display increased resistance to CHG

The minimum inhibitory concentration of CHG was determined for isolates from the *Bacillus, Corynebacterium, Enterococcus, Micrococcus, Staphylococcus,* and *Pseudomonas* genera cultured at baseline and follow-up visits. Consistent with the shifts in the microbial community composition, <200 µg/mL of CHG was sufficient to prevent the growth of *Corynebacterium, Cutibacterium, Micrococcus,* and *Staphylococcus* (including *S. aureus* and *CoNS Staphylococci*) species (Table 2). However, isolates of *Bacillus* and *Enterococcus* displayed increased resistance to CHG, with MICs ranging from 250 to 1,250 µg/mL. Epidemiologic MIC cut-off values are species-specific MIC limits based on the intrinsic CHG tolerance of wild-type isolates before the acquisition of mutations that confer additional protection (34)*. E. faecalis, S. aureus, and S. epidermidis* isolates all showed MICs higher than their proposed species-specific epidemiologic cut-offs (64 µg/mL for *E. faecalis;* 8–16 µg/mL for *S. aureus*; 4 µg/mL for *S. epidermisis*), revealing that these isolates may be more resistant to CHG than anticipated (34–39). Collectively, these findings suggest that most individuals likely have some degree of antiseptic resistance within their skin microbial communities, and some may harbor highly resistant taxa even prior to antiseptic exposure.

**TABLE 2:** *Bacillus* and *Enterococcus* isolates display increased resistance to CHG^*a*^

Isolate	LK-ID	Subject	Collection timepoint	Site	Average MIC (µg/mL)
*Bacillus safensis*	LK2236	CHG-002	Baseline	Control ite	250
*Bacillus* spp. 1	LK3019	CHG-025	Baseline	Control site	500
*Bacillus* spp. 2	LK3063	CHG-028	Baseline	Control site	1250
*Corynebacterium mycetoides*	LK2860	CHG-021	Baseline	Control site	31
*Corynebacterium sanguinis* 1	LK2319	CHG-007	Baseline	Control site	12
*Corynebacterium sanguinis* 2	LK2685	CHG-017	Baseline	Control site	47
*Corynebacterium striatum*	LK2376	CHG-003	Follow-Up	Surgical site	63
*Corynebacterium ureicelerivorans*	LK3068	CHG-028	Baseline	Control site	31
*Cutibacterium* spp.	LK2346	CHG-010	Baseline	Control site	47
*Dietzia* spp.	LK2320	CHG-008	Baseline	Control site	16
*Enterococcus faecalis*	LK2699	CHG-006	Follow-up	Surgical site	750
*Micrococcus endophyticus*	LK2945	CHG-018	Baseline	Control site	16
*Micrococcus luteus* 1	LK2435	CHG-012	Baseline	Surgical site	63
*Micrococcus luteus* 2	LK2473	CHG-014	Baseline	Control site	31
*Micrococcus luteus* 3	LK2281	CHG-005	Baseline	Control site	125
*Micrococcus yunnanensis* 1	LK2439	CHG-012	Baseline	Surgical site	63
*Micrococcus yunnanensis* 2	LK2686	CHG-017	Baseline	Control site	24
*Pseudomonas luteola*	LK2294	CHG-007	Baseline	Control site	125
*Staphylococcus aureus*	LK2886	CHG-016	Follow-Up	Control site	94
*Staphylococcus capitis*	LK2681	CHG-016	Baseline	Control site	63
*Staphylococcus epidermidis* 1	LK2219	CHG-001	Baseline	Control site	187.5
*Staphylococcus epidermidis* 2	LK2279	CHG-006	Baseline	Control site	73
*Staphylococcus hominis* 1	LK2452	CHG-012	Baseline	Surgical site	94
*Staphylococcus hominis* 2	LK2226	CHG-003	Baseline	Control site	94
*Staphylococcus* spp. 1	LK2887	CHG-016	Follow-Up	Control site	146
*Staphylococcus warneri* 1	LK2474	CHG-012	Baseline	Surgical site	47
*Staphylococcus warneri* 2	LK2894	CHG-016	Baseline	Control site	47

^*a*^ Bacterial Isolates collected from surgical subjects were identified via Sanger sequencing of the 16S rRNA gene. The minimum concentration of CHG needed to inhibit isolate growth was measured via a disk diffusion MIC assay. Isolates were tested in duplicate, and the average MIC was calculated. This table also includes the subject and sample from which each isolate was collected.

## DISCUSSION

Surgical site infections continue to occur despite the widespread adoption of surgical antiseptics, including CHG. However, the impacts on the healthy skin microbiome remain poorly studied. Here, we characterize the immediate and long-term effects of CHG on the absolute burden and microbial composition of viable skin bacteria in patients undergoing elective surgeries. Our findings demonstrate that CHG i) effectively reduces viable microbial bioburden at the surgical site and ii) selects for several potentially pathogenic taxa, particularly gram-negative bacteria. We also demonstrate that skin microbiomes recover, indicating that changes are transient and the skin microbiome itself is resilient to these insults. In short, these findings underscore that broad-scale application of topical antiseptics like CHG may disrupt healthy skin microbiota, resulting in space for others to proliferate, particularly pathogenic gram-negative and biofilm-forming taxa.

CHG has a unique ability to bind any DNA to the skin surface (22), resulting in inconsistent results using molecular methods (21–25). To circumvent DNA from newly killed bacteria confounding sequencing results, we optimized a viability assay for selective evaluation of DNA from live microorganisms within the skin microbiome. With this method, we show that under normal circumstances, approximately 60%–80% of bacterial DNA on the skin is from viable microbes within the community. This stands in contrast to the recent findings reported by Acosta *et al*. that bacterial DNA on the skin surface overrepresents the viable skin microbiome (27). Too high a concentration of PMA or PMAxx for the sample biomass can result in false-negatives, either due to being toxic to live cells or PMA/PMAxx remaining active beyond the duration of photoactivation and subsequent interaction with DNA released during the extraction process from live cells (40). In tandem with our observation that 50 µM PMAxx negatively interacts with the low biomass of skin microbial communities**,** it is possible that Acosta *et al*.’s use of 50 µM PMA was cytotoxic, resulting in a misrepresentation of the proportion of DNA from dead microbes on the skin.

Significant differences between the viable and total microbial bioburden and community composition on the day of surgery support that CHG can bind to and retain non-viable microbial DNA at the skin surface (22). We posit that following antiseptic application, free DNA from newly killed bacteria, particularly from highly abundant skin commensal taxa, persists on the skin after CHG application and masks true microbial community shifts. Our findings underscore the importance of assessing the viable portion of the microbiome in this and similar contexts of antiseptic, antibiotic, or other microbially toxic exposure studies.

We confirm that pre-surgical preparation with CHG drastically reduces viable microbial bioburden. Importantly, we also reveal that CHG alters viable microbial community compositions, particularly at the surgical site. On the day of surgery, these communities display reduced relative abundance of several commensal skin taxa (e.g., *Corynebacterium, Micrococcus,* and coagulase-negative *Staphylococcus* (CoNS) species). Skin commensals help maintain community homeostasis in part through microbe–microbe interactions that prevent pathogen overgrowth (7, 41–45). The relative loss of numerous commensal taxa following CHG may result in a skin microbiome impaired in its ability to defend against opportunistic pathogens.

Some bacterial species can intrinsically tolerate higher levels of CHG (34). Baseline microbiomes harboring microbes with higher intrinsic antiseptic tolerance and/or acquired antiseptic resistance genes likely influence community dynamics following pre-surgical preparations. We reveal that some individuals harbor highly resistant taxa prior to pre-surgical antiseptic exposure. Overall, our findings are consistent with multiple reports that CHG is less effective against gram-negative bacteria and microbes within a biofilm (14, 21, 46–48*). Staphylococcus aureus, S. epidermidis, Escherichia coli,* and *Pseudomonas aeruginosa* are among the most common causes of SSI (1). Our findings are also consistent with reports that there is increasing isolation of strains resistant to CHG (34, 36, 49–51). Incomplete and diminishing efficacy of CHG against these taxa may partially explain why these pathogens continue to commonly cause SSI.

Reassuringly, most subjects’ viable microbial bioburden and community composition resembled baseline at their follow-up clinic visit. Although we were unable to interrogate precisely when skin microbial communities repopulate, based on data from ethanol- and povidone-iodine-based antiseptics (22, 52), microbial bioburden likely starts to recover within hours. Sources of repopulation likely include microbes residing deep within hair follicles (53), neighboring body sites that received a lower dose of CHG, as well as patients’ own clothes and home environment (7, 54). Together, these findings reinforce the resiliency of skin microbial communities to acute perturbations and support that for individuals preparing for surgery, the effects of CHG antiseptic on skin microbial communities are temporary.

One of the main limitations of this study was limited enrollment to elective outpatient surgeries. To address this, future works aim to expand this study design to more surgery types, particularly surgeries requiring post-operative inpatient stays. Another limitation is the taxonomic resolution afforded by 16S rRNA gene sequencing. Metagenomic shotgun sequencing would provide a more comprehensive insight into strain-level, multi-kingdom, and functional impacts of CHG on the skin. Metagenomic sequencing could also identify antimicrobial resistance genes and genes associated with enhanced CHG resistance.

In conclusion, we confirm that pre-surgical application of CHG does reduce viable skin microbial bioburden. However, complete sterility is not achieved. Chlorhexidine induces a temporary shift in the microbial composition. Skin microbial communities at the time of surgery are enriched for several potentially pathogenic taxa, notably *Pseudomonas, Escherichia, Acinetobacter,* and *Bacillus*. Incomplete antiseptic efficacy against these taxa likely places some patients at disproportionate risk for developing an SSI. Importantly, with this work, we have addressed a critical uncertainty in the field (21–25). To our knowledge, this is the first work to accurately demonstrate the impact of CHG on viable, rather than total, skin microbial bioburden and community composition via sequencing-based methods. Collectively, our findings identify tangible avenues to improve antiseptic formulations and pre-surgical preparation protocols to better target gram-negative and biofilm-forming microbes while protecting beneficial skin commensals. With these efforts, we can further reduce the burden of surgical site infections.

## Data Availability

Sequence reads for this project can be found under NCBI BioProject PRJNA1092813. Code for analysis and generation of figures can be found on GitHub at https://github.com/Kalan-Lab/Townsend_etal_StillNotSterile.

## SUPPLEMENTAL MATERIAL

Supplemental figures part 1 spectrum.02873-24-s0001.pdf Figures S1-S4.

Supplemental figures part 2 spectrum.02873-24-s0002.pdf Figures S5-S7.

Supplemental figures part 3 spectrum.02873-24-s0003.pdf Figures S8 and S9.

Supplemental tables spectrum.02873-24-s0004.xlsx Tables S1-S8.

ASM does not own the copyrights to Supplemental Material that may be linked to, or accessed through, an article. The authors have granted ASM a non-exclusive, world-wide license to publish the Supplemental Material files. Please contact the corresponding author directly for reuse.
