# Supplementary material for: Still not sterile: viability-based assessment of the skin microbiome following pre-surgical application of a broad-spectrum antiseptic reveals transient pathogen enrichment and long-term recovery
Source: Microbiol Spectr. 2025 Apr 10;13(5):e02873-24. doi: 10.1128/spectrum.02873-24 (PMC12054058; doi:10.1128/spectrum.02873-24)
Supplement: Supplemental figures part 1 — Figures S1-S4. [file spectrum.02873-24-s0001.pdf]

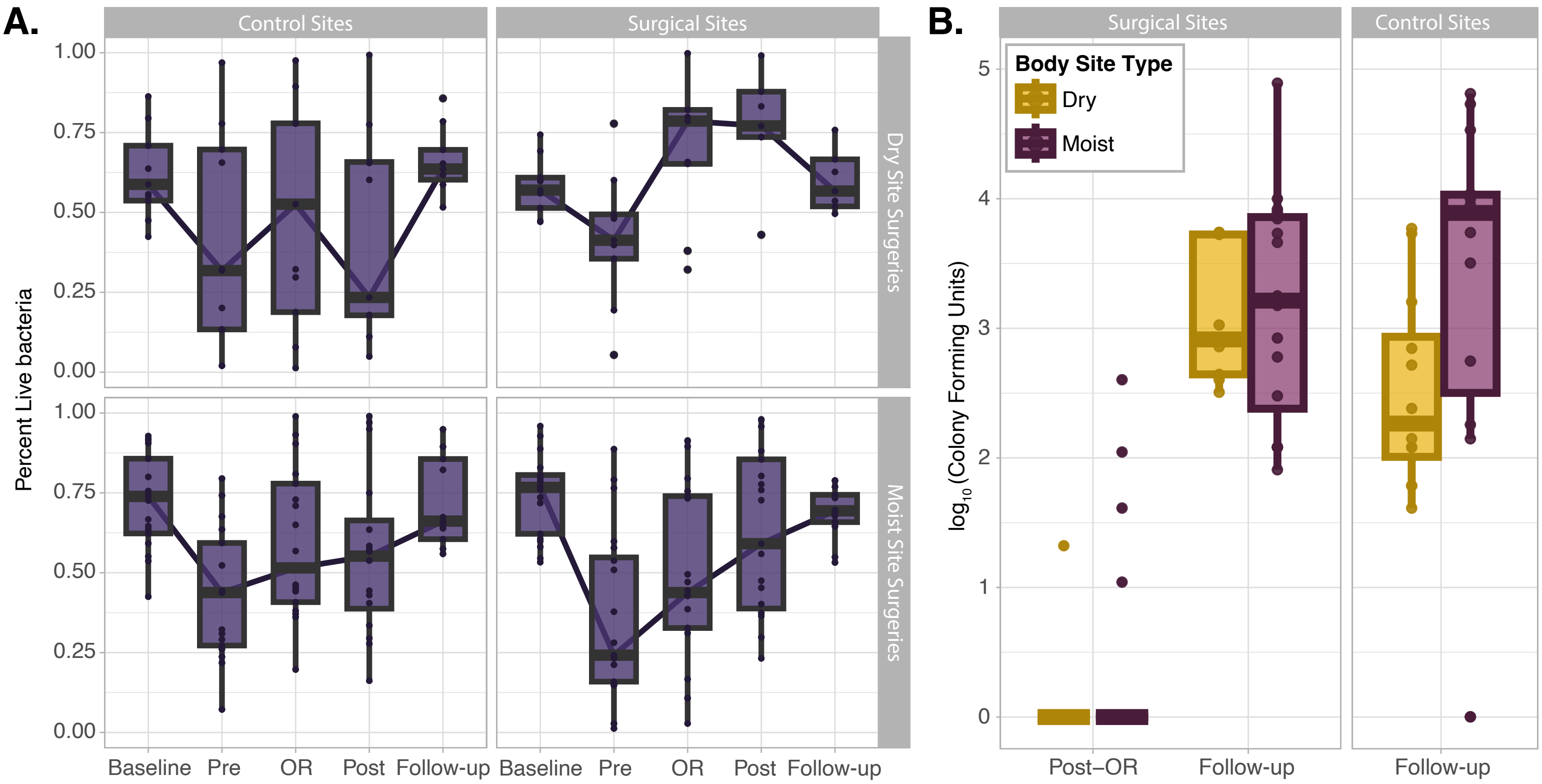

Supplemental Figure 1: Viable Microbial bioburden. A. Companion figure to **Figure 3A.** Points represent the percent live bacteria within each subject's control and surgical site samples over time. Data also grouped by whether the sample was from a moist or dry body site. **B**. Swabs of the skin microbiome at subjects' surgical sites in the post-operative care unit and from both the control and surgical site during the post-surgery clinic visit. Plot displays the culturable bacterial bioburden at moist and dry sampling sites at both timepoints.

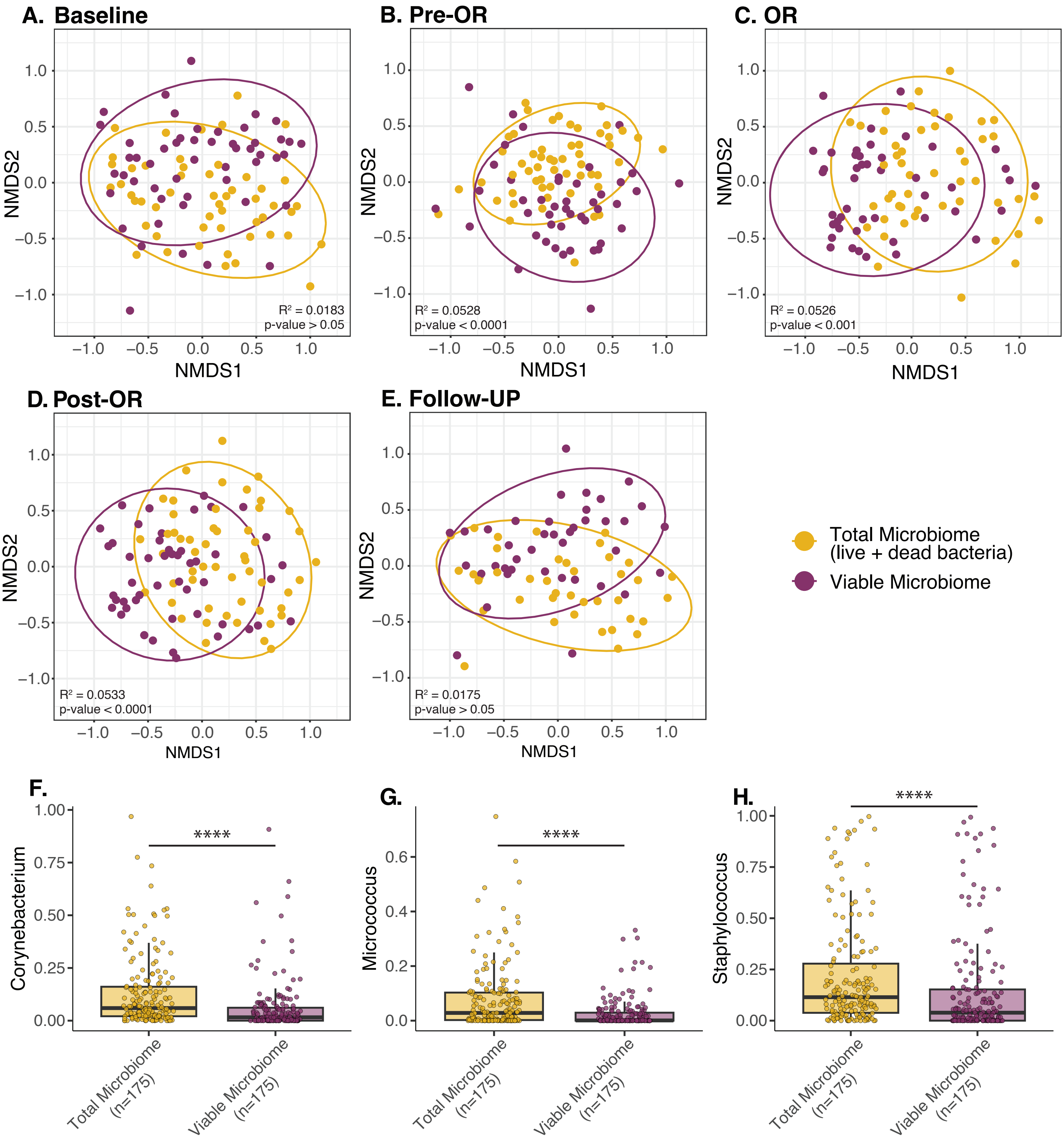

Supplemental Figure 2: Viable and total microbial community compositions differ on the day of surgery. A-E. Non-metric Multidimensional Scaling (NMDS) ordination of the Bray-Curtis beta-diversity at each time-point. PERMANOVAs with 9999 permutations were utilized to evaluate the differences between the viable (PMAxx treated) and total (not treated) sample community compositions. Details can be found in **supplemental table 4. F-H.** MAASLIN2 was used to determine differences in the relative abundance of individual taxa between viable and total communities from samples collected on the day of surgery (Pre-OR, OR, and Post-OR timepoints combined). **** indicates FDR q-value with Benjamini-Hochberg correction < 0.0001.

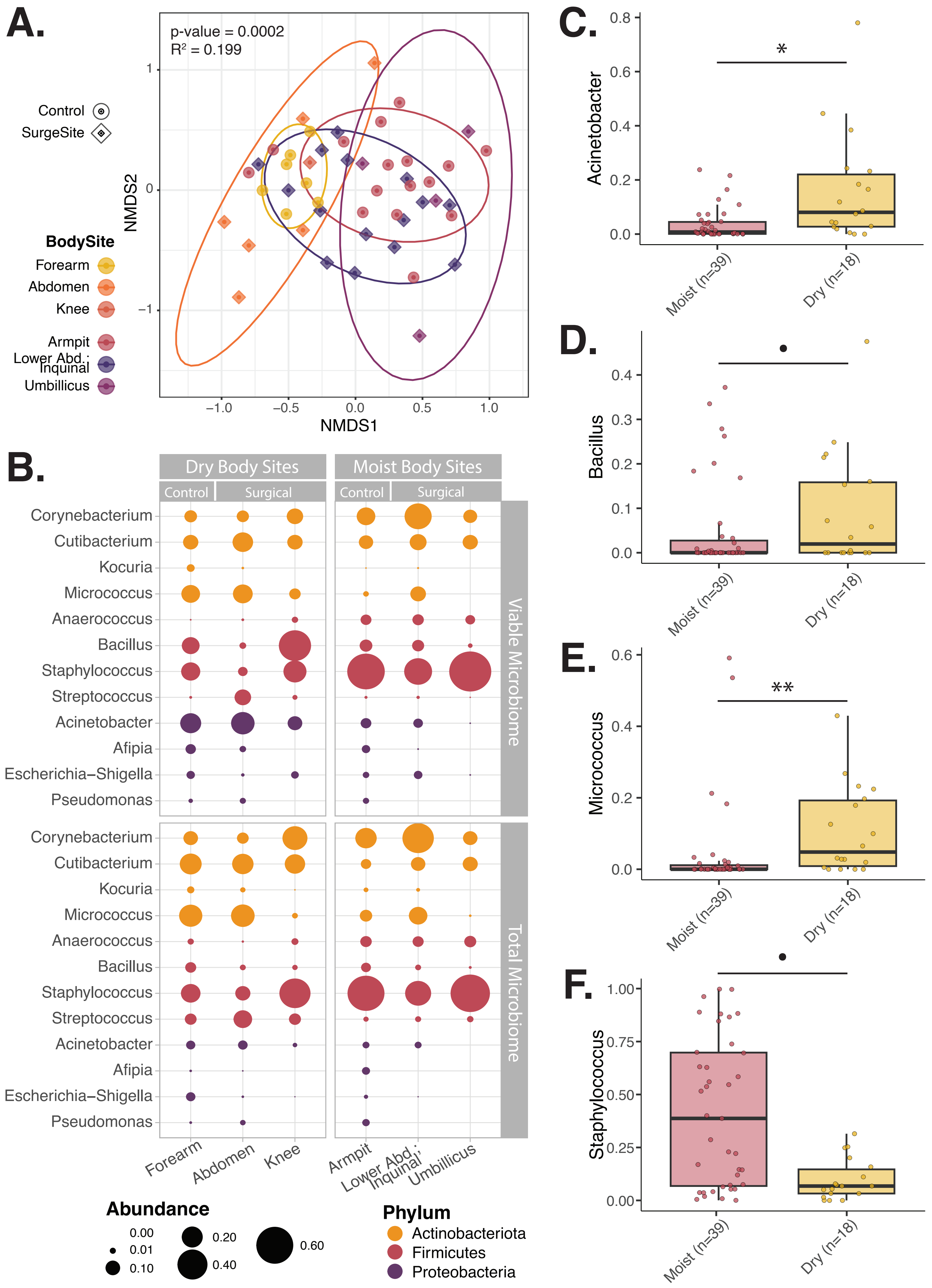

A.
p-value = 0.0002
R² = 0.199
Control
SurgeSite
BodySite
Forearm
Abdomen
Knee
Armpit
Lower Abd.; Inquinal
Umbillicus
NMDS1
NMDS2
B.
Dry Body Sites
Moist Body Sites
Control
Surgical
Corynebacterium
Cutibacterium
Kocuria
Micrococcus
Anaerococcus
Bacillus
Staphylococcus
Streptococcus
Acinetobacter
Afipia
Escherichia–Shigella
Pseudomonas
Viable Microbiome
Total Microbiome
Forearm
Abdomen
Knee
Armpit
Lower Abd.; Inquinal
Umbillicus
Abundance
0.00
0.01
0.10
0.20
0.40
0.60
Phylum
Actinobacteriota
Firmicutes
Proteobacteria
C.
Acinetobacter
*
D.
Bacillus
E.
Micrococcus
**
F.
Staphylococcus
Moist (n=39)
Dry (n=18)

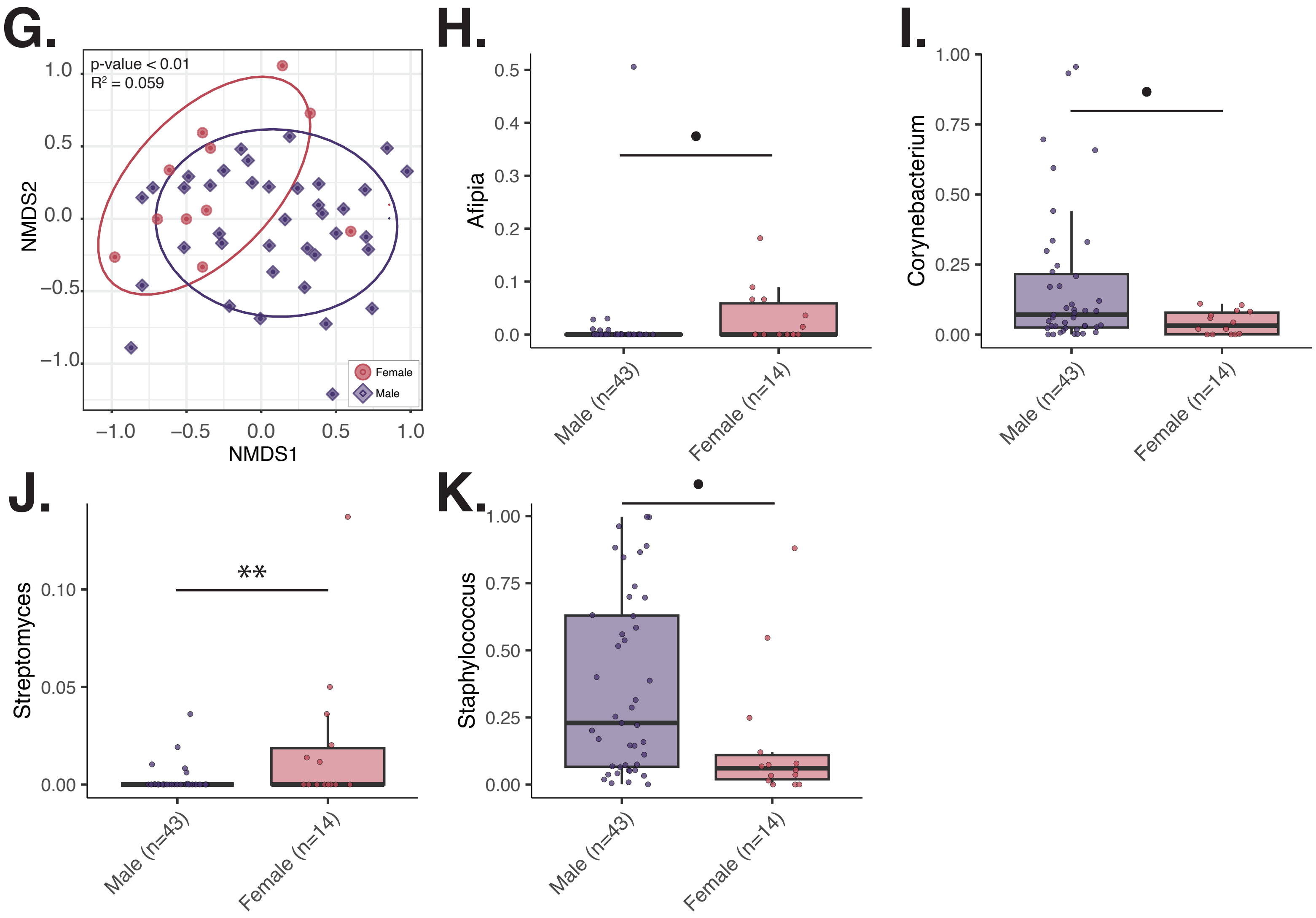

Supplemental Figure 3: Microbial community composition at baseline is associated with body site of sample collection and subject gender. A. NMDS ordination of Bray-Curtis beta-diversity of viable microbial communities at subjects' baseline clinic visit highlighting the association between the microbiome composition and body site of sample collection (univariate PERMANOVA with 9999 permutations). **B.** plot of average relative abundance of key genera within viable (top row) and total (bottom row) microbial communities across body sites samples collected at the baseline timepoint. Size of the dot indicates the average genera relative abundance. **C-F.** MAASLIN2 was used to determine differences in the relative abundance of individual taxa within viable communities of samples collected at moist body sites (armpit, lower abdomen to inguinal, and umbilicus combined) compared to those from dry body sites (forearm, central abdomen, and knee combined). Both control and surgical site samples were included in this analysis. In these calculations both subject and gender were incorporated as random effects. Only taxa with significantly different relative abundance (p-value < 0.05) between the groups are shown. FDR q-values with Benjamini-Hochberg correction indicated. **G.** Bray-Curtis beta diversity NMDS ordination highlighting the association of subject gender with baseline viable microbial community composition (univariate PERMANOVA with 9999 permutations). **H-K.** Differences in the relative abundance of individual taxa within the viable baseline microbiome of male and female subjects were assessed via MAASLIN2. Both control and surgical site samples were included in this analysis and subject and body site of sample collection were incorporated as random effects into these calculations. Only taxa with significantly different relative abundance (p-value < 0.05) between the groups are shown. FDR q-values with Benjamini-Hochberg correction indicated.

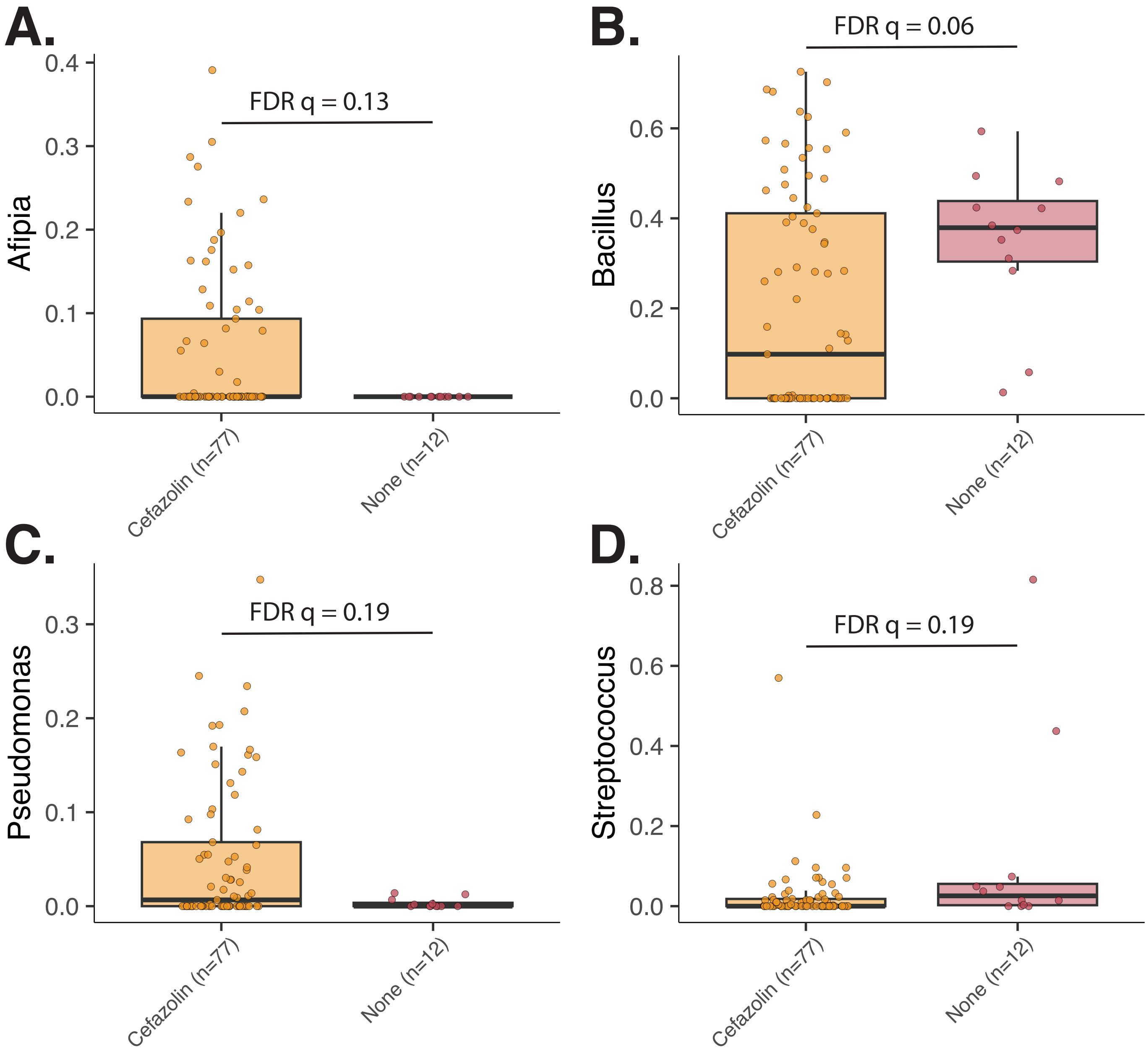

Supplemental Figure 4: Antibiotic prophylaxis with cefazolin is associated with reduced relative abundance of Bacillus and Streptococcus and increased Afipia and Pseudomonas at the surgical site on the day of surgery. A-D. Viable microbiome samples from the surgical site on the day of surgery, the samples collected at the pre-OR, OR, and post-OR timepoints, were assessed collectively. Differences in taxa relative abundance were evaluated with MAASLIN2 incorporating the individual subject, gender, and body site of sample collection as random-effects. Only taxa with significantly different relative abundance (p-value < 0.05) between the groups are shown. FDR q-values with Benjamini-Hochberg correction indicated.
