# Supplementary material for: Still not sterile: viability-based assessment of the skin microbiome following pre-surgical application of a broad-spectrum antiseptic reveals transient pathogen enrichment and long-term recovery
Source: Microbiol Spectr. 2025 Apr 10;13(5):e02873-24. doi: 10.1128/spectrum.02873-24 (PMC12054058; doi:10.1128/spectrum.02873-24)
Supplement: Supplemental figures part 2 — Figures S5-S7. [file spectrum.02873-24-s0002.pdf]

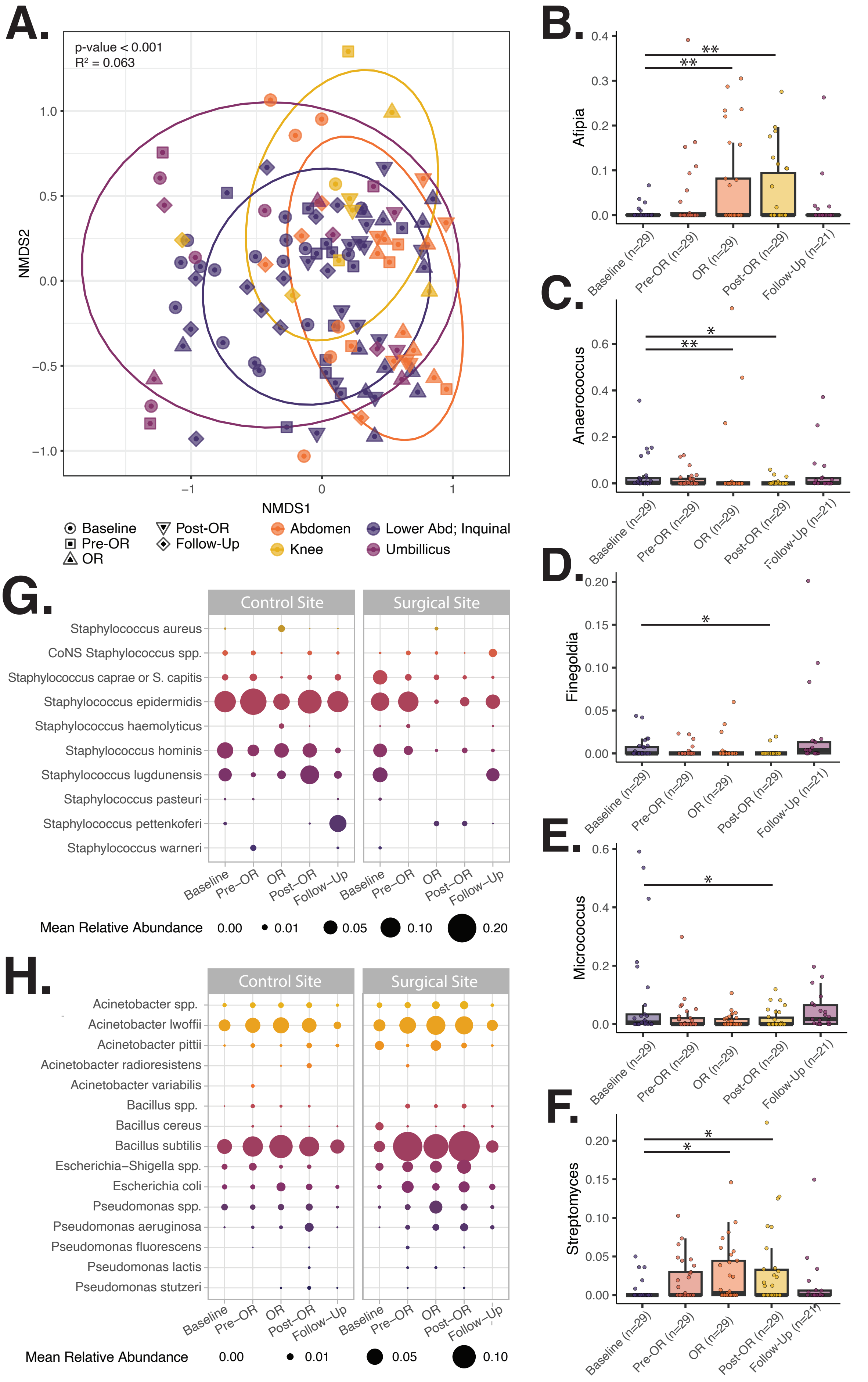

Supplemental Figure 5: Exposure to Chlorhexidine Gluconate is associated with changes in viable microbial community composition at the surgical site on the day of surgery. Continuation of **Figure 4. A.** Bray-Curtis beta-diversity NMDS ordination highlighting the association between viable microbial community composition and the body location for the surgical site (univariate PERMANOVA with 9999 permutations). Details can be found in **supplemental table 7. B-F.** Relative abundance plots for taxa significantly more or less abundant in the viable microbiome at the surgery site on the day of surgery, after exposure to CHG, compared to the baseline sample collection. Differential abundance of taxa at each later timepoint compared to the baseline timepoint were assessed via MAASLIN2 and evaluations were made accounting for the individual subject, subject gender, body site of sample collection, and antibiotic prophylaxis as random effects. **Supplemental table 8** contains further details for these results. Note; one subject underwent simultaneous umbilical and inguinal hernia repair. Both sites were sampled at all timepoints. Thus the n = 29 at the baseline through Post-OR timepoints and n=21 at follow-up, which is one more than the total number of subjects who underwent surgery and came for in-person follow-up, n = 28 and = 20 respectively. **G.** ASVs from the Staphylococcus genus were aligned against the BLAST database to obtain probable species assignment. Plot of mean relative abundance of Staphylococcus species within viable microbial communities over time. Apart from S. aureus, all species identified are coagulase negative Staphylococci (CoNS). “CoNS Staphylococcus spp.” indicates ASVs that aligned well to several CoNS species. **H.** ASVs from the Acinetobacter, Bacillus, Escherichia-shigella, and Pseudomonas genera were aligned against the BLAST database to obtain probable species assignment. Plot displays mean relative abundance of these species within viable microbial communities over time.

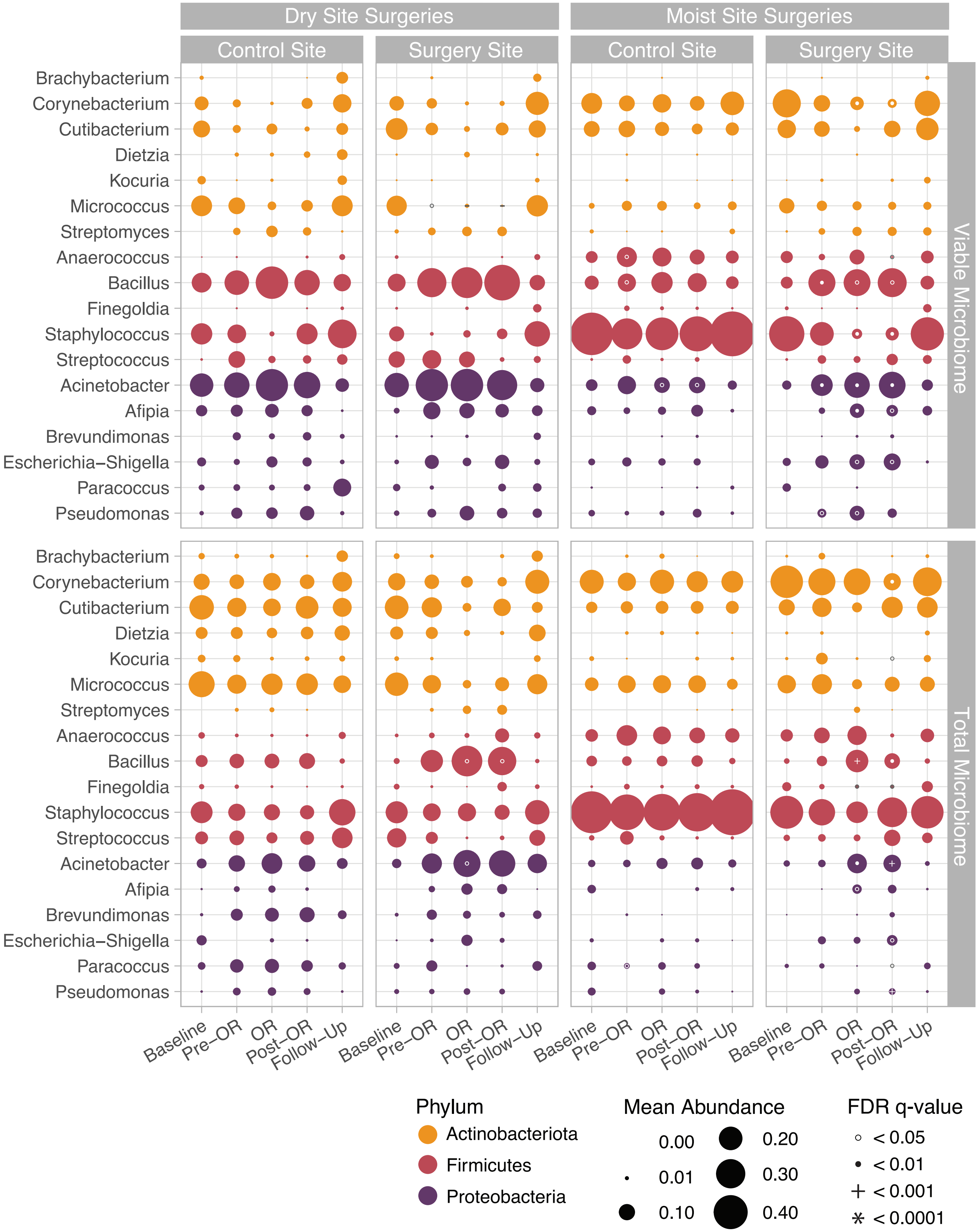

Supplemental Figure 6: Change in average relative abundance of key taxa over time at surgical and control sites collected from most and dry body sites. Companion to **Figure 4C** displaying the average relative abundance of each taxa over time in the viable and total microbial communities at moist and dry surgical sites and control sites respectively. Mean taxa abundance is indicated by the size of the point. Differential relative abundance of taxa at later timepoints versus at baseline was evaluated via MAASLIN2. All MAASLIN evaluations were made accounting for subject, body site of sample collection, gender, and antibiotic prophylaxis as random effects. White, or in a few cases grey, circles, filled-in dots, plus sign, and asterixis indicate the degree of significance. Further details for the MAASLIN2 results located in **Supplemental Table 8.**

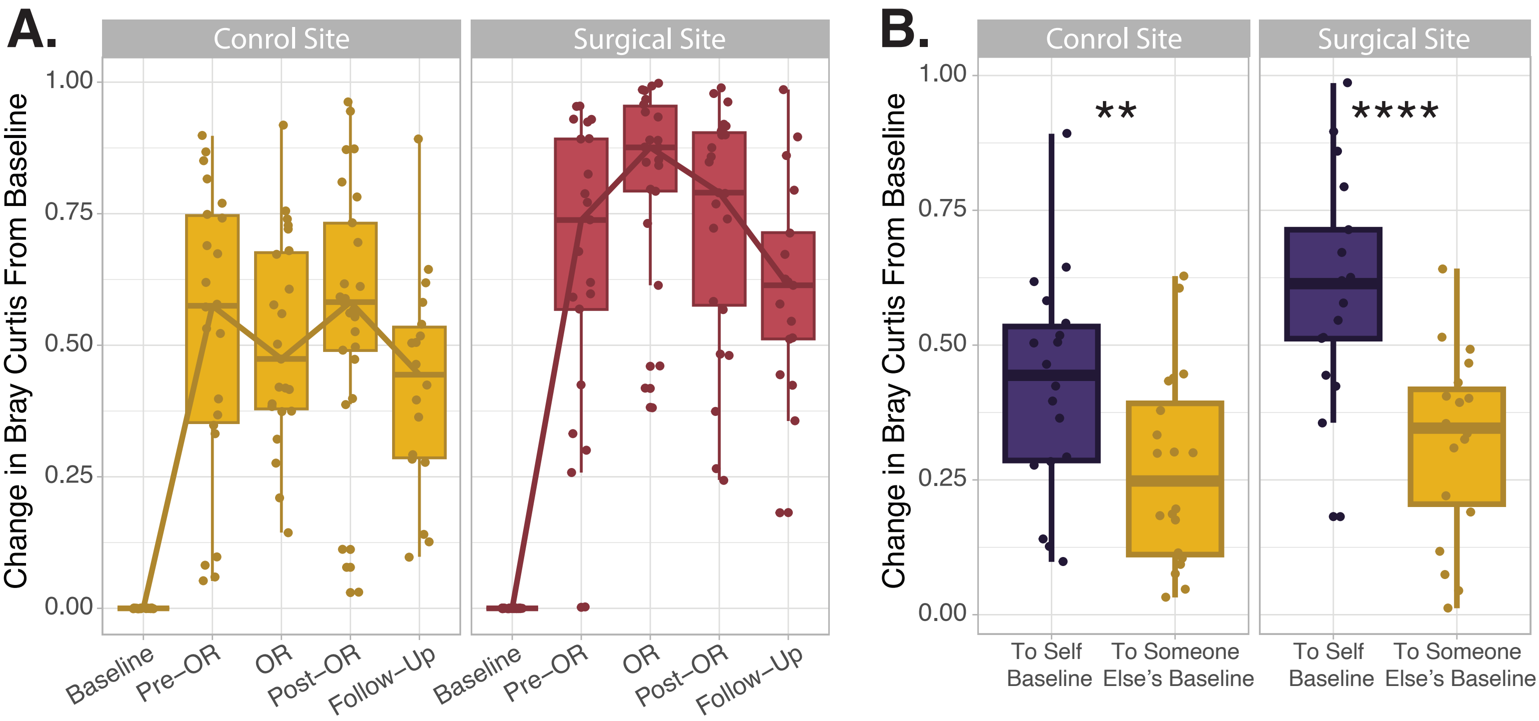

Supplemental Figure 7: Change microbial community composition from baseline. A. Similarity of each subject's surgical or control site microbiome at each timepoint was compared to their respective baseline community composition via the Bray-Curtis beta diversity metric. **B.** Bray Curtis beta diversity metric was also used to evaluate the similarity of each subject's microbiome composition at follow-up to both their own baseline microbiome composition and the most similar baseline microbial community composition (smallest Bray-Curtis distance) of another subject. Differences between the bray-curtis distance between a subject's follow-up to their own baseline versus someone elses baseline microbiome were evaluated with the Wilcoxon matched pairs signed rank tests. ** indicates p-value < 0.01; **** indicates p-value < 0.0001.
