# Supplementary material for: Still not sterile: viability-based assessment of the skin microbiome following pre-surgical application of a broad-spectrum antiseptic reveals transient pathogen enrichment and long-term recovery
Source: Microbiol Spectr. 2025 Apr 10;13(5):e02873-24. doi: 10.1128/spectrum.02873-24 (PMC12054058; doi:10.1128/spectrum.02873-24)
Supplement: Supplemental figures part 3 — Figures S8 and S9. [file spectrum.02873-24-s0003.pdf]

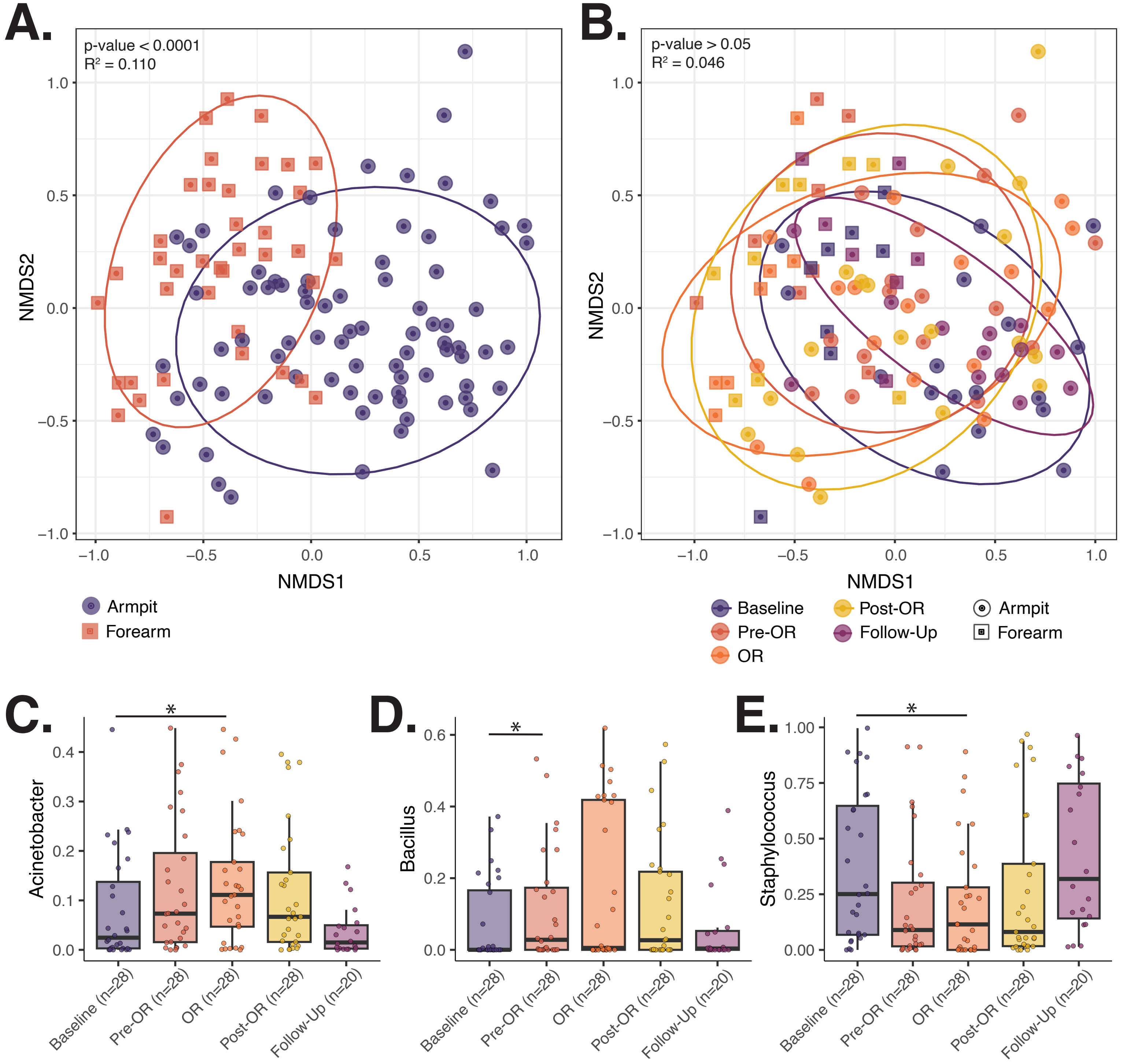

Supplemental Figure 8: Viable communities at the control sites are more associated with the body site of sample collection. Companion figure to **figure 4** and **supplemental figure 5**. **A.** Bray-Curtis beta diversity NMDS ordination illustrating the strong association of body site of control samples with viable microbial community composition. **B**. Bray-Curtis beta-diversity ordination highlighting the lack of association between viable microbial community composition and timepoint of control site sample collection. Associations of microbial community composition with various features were evaluated via univariate PERMANOVAs with 9999 permutations. Details can be found in **supplemental table 7. C-E.** Relative abundance of Acinetobacter (C), Bacillus (D), and Staphylococcus (E) in viable communities at both moist (armpit) and dry (forearm) control sites over time. Differential abundance of taxa at each timepoint compared to the baseline timepoint were assessed via MAASLIN2. All MAASLIN2 evaluations were made accounting for individual subjects, gender, body site of sample collection and antibiotic prophylaxis as random effects.

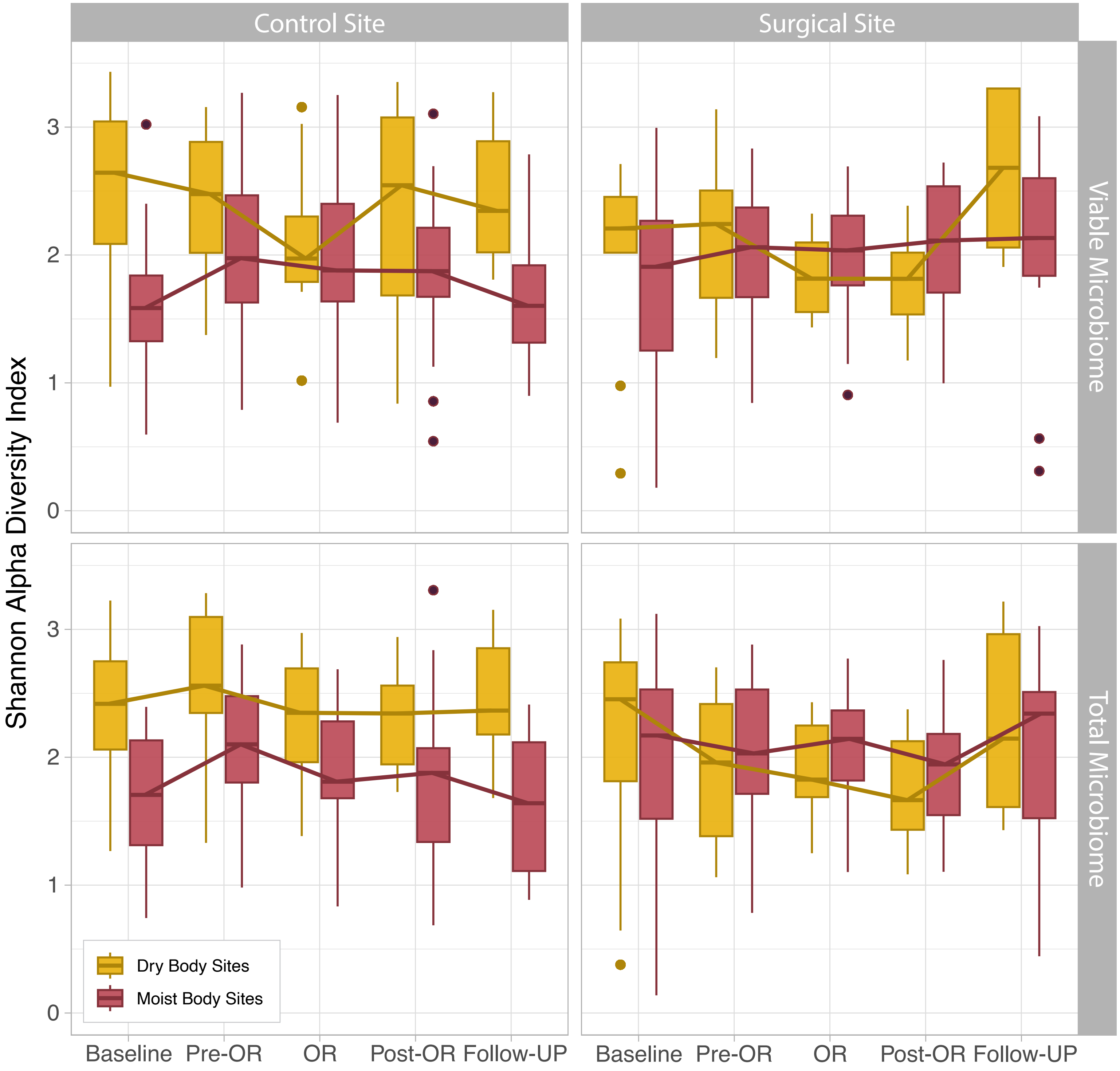

Supplemental Figure 9: No change in Shannon alpha diversity following CHG application. Shannon alpha diversity metric was used to measure the microbial diversity within each sample. Boxplots represent the Shannon index distribution (median interquartile range) of viable and total microbial communities at the surgical and control sites. Samples are grouped by if they were collected at a moist or dry body site.
